# Epigenetic Heritability of Cell Plasticity Drives Cancer Drug Resistance through a One-to-Many Genotype-to-Phenotype Paradigm

**DOI:** 10.1158/0008-5472.CAN-25-0999

**Published:** 2025-06-11

**Authors:** Erica A. Oliveira, Salvatore Milite, Javier Fernandez-Mateos, George D. Cresswell, Erika Yara-Romero, Georgios Vlachogiannis, Bingjie Chen, Chela T. James, Lucrezia Patruno, Gianluca Ascolani, Ahmet Acar, Timon Heide, Inmaculada Spiteri, Alex Graudenzi, Giulio Caravagna, Andrea Bertotti, Trevor A. Graham, Luca Magnani, Nicola Valeri, Andrea Sottoriva

**Affiliations:** 1 Centre for Evolution and Cancer, The Institute of Cancer Research, London, United Kingdom.; 2 Computational Biology Research Centre, Human Technopole, Milan, Italy.; 3 St. Anna Children’s Cancer Research Institute, Vienna, Austria.; 4 GMU-GIBH Joint School of Life Sciences, The Guangdong-Hong Kong-Macau Joint Laboratory for Cell Fate Regulation and Diseases, Guangzhou Medical University, Guangzhou, China.; 5 Department of Informatics, Systems and Communication, University of Milan-Bicocca, Milan, Italy.; 6 Department of Biological Sciences, Middle East Technical University, Ankara, Turkey.; 7 Department of Mathematics, Informatics and Geosciences, University of Trieste, Trieste, Italy.; 8 Department of Oncology, University of Torino, Turin, Italy.; 9 Candiolo Research Institute – FPO IRCSS, Turin, Italy.; 10 Division of Breast Cancer Research, The Institute of Cancer Research, London, United Kingdom.; 11 Department of Surgery and Cancer, Imperial College London, London, United Kingdom.

## Abstract

Cancer drug resistance is multifactorial, driven by heritable (epi)genetic changes but also by phenotypic plasticity. In this study, we dissected the drivers of resistance by perturbing organoids derived from patients with colorectal cancer longitudinally with drugs in sequence. Combined longitudinal lineage tracking, single-cell multiomics analysis, evolutionary modeling, and machine learning revealed that different targeted drugs select for distinct subclones, supporting rationally designed drug sequences. The cellular memory of drug resistance was encoded as a heritable epigenetic configuration from which multiple transcriptional programs could run, supporting a one-to-many (epi)genotype-to-phenotype map that explains how clonal expansions and plasticity manifest together. This epigenetic landscape may ensure drug-resistant subclones can exhibit distinct phenotypes in changing environments while still preserving the cellular memory encoding for their selective advantage. Chemotherapy resistance was instead entirely driven by transient phenotypic plasticity rather than stable clonal selection. Inducing further chromosomal instability before drug application changed clonal evolution but not convergent transcriptional programs. Collectively, these data show how genetic and epigenetic alterations are selected to engender a “permissive epigenome” that enables phenotypic plasticity.

**Significance:**

Drug resistance is driven by genetic–epigenetic memory that enables cancer cells to adopt multiple phenotypic states depending on environmental conditions, supporting integration of evolutionary principles into biomarker discovery and personalized treatment strategies.

This article is part of a special series: Driving Cancer Discoveries with Computational Research, Data Science, and Machine Learning/AI.

## Introduction

Cancers develop following the Darwinian rules of clonal evolution, in which selected subclones bearing new heritable alterations come to dominate the cellular compartment (1). The same paradigm is extended to explain the emergence of treatment resistance, arguably the biggest problem in oncology today (2). However, only a subset of resistance mechanisms has been identified (3–5). Even when genetic mutations are known to cause resistance, often these are only detected in a minor proportion of cells within tumors that are refractory to treatment. In many patients, treatment failure remains entirely unexplained by genetic alterations alone. Accumulating evidence suggests that additional Darwinian mechanisms involving nongenetic alterations (3, 6, 7), as well as non-Darwinian cellular plasticity (8), also contribute to tumorigenesis. Importantly, these mechanisms can coexist in the same tumor at the same time; hence, therapy resistance is likely multifaceted (9).

Relatively little attention has been paid to identifying epigenetic changes that drive cancer evolution, and the characterization of “epigenetic drivers” has been hindered by the lack of proper controls to compare cancer and normal epigenomes from the same tissue of origin (10). Further, because cancer cell plasticity is inherently a dynamic property, it is challenging to study in clinical samples typically collected at a single time point (11). Elegant experiments based on the Luria–Delbrück approaches have demonstrated in cell lines that indeed one can distinguish plasticity from Darwinian adaptive changes (12). Such mechanisms have also been identified in patient-derived xenograft models under the pressure of anti-EGFR drugs (13).

Fundamental open questions remain, such as “To what extent are epigenetic changes heritable upon cell division?” “Is plasticity a new cancer program or a reactivated cellular state?” “Is plasticity reversible?” and “Is the propensity for cell plasticity a heritable trait in itself?” As therapy resistance is multifactorial, another important question is, “Are different subclones in the same tumor adapting differently to drugs?” Answering these complex questions requires concomitant measurements of genomes, epigenomes, and transcriptomes, matched with cell lineage histories to deconvolute Darwinian from non-Darwinian mechanisms. Because drug resistance is multifactorial in the same tumor, single-cell resolution is also required.

In this study, we designed an evolutionary experiment using patient-derived tumor organoids under the pressure of different sequences of drugs. We used expressed lentiviral barcodes combined with single-cell multiomics to track the evolution of single-cell genomes, epigenomes, and transcriptomes over time. We studied adaptation in persister cells under the pressure of drugs and in the same cell lineages when drug pressure was released to understand how the therapeutic environment dynamically perturbs cells. We leveraged archetype analysis to chart the mapping between data modalities in individually tracked subclones.

## Materials and Methods

### Organoid culture and passaging

Microsatellite stable (MSS) F-016 patient-derived organoid (PDO) was established from a liver metastasis of a colorectal tumor, and it has been previously characterized as described in Vlachogiannis and colleagues (14). Among its pathogenic mutations, *AKT1* c.49G>A_p.Glu17Lys and *AKT1* amplification were of interest for our purpose as this organoid was a good model of oncogenic addiction, showing high sensitivity and selective apoptosis to Akt inhibitors. CRC0282 PDO was established from the cohort in ref. 13. *Mycoplasma* was periodically tested during the culture of organoids. Written informed consent was obtained from patients. Studies were conducted in accordance with the Declaration of Helsinki. Studies were approved by an Institutional Review Board (Prospect C: National Research Ethics Service: 12/LO/0914; Prospect R: Medicines and Healthcare products Regulatory Agency: 15983/0249/001–0001).

PDOs were cultured embedded in Growth Factor Reduced Basement Membrane Matrix (Corning), hereinafter referred to as Matrigel, and Advanced DMEM/F-12 media (Thermo Fisher Scientific), supplemented with 1× B27 and 1× N2 supplements (Thermo Fisher Scientific), 0.01% BSA (Roche), 2 mmol/L L-glutamine (Thermo Fisher Scientific), and 100 U/mL penicillin/streptomycin (Thermo Fisher Scientific). Additionally, 12 different growth factors were used to maintain PDO culture: 50 ng/mL EGF, 100 ng/mL Noggin, 500 ng/mL R-spondin 1, 10 ng/mL FGF-basic, and 10 ng/mL FGF-10 (all from PeproTech); 10 nmol/L gastrin, 10 µmol/L Y-27632, 4 mmol/L nicotinamide, and 5 µmol/L SB202190 (all from Sigma-Aldrich); 100 ng/mL Wnt-3a (R&D Systems); and 1 µmol/L prostaglandin E_2_ and 0.5 µmol/L A83-01 (Tocris Bioscience).

Passaging of PDOs was performed using TrypLE 1× diluted in 1 mmol/L PBS-EDTA (Thermo Fisher Scientific). In short, after media removal, PDOs in Matrigel were harvested by pipetting with 1 mL of TryplE 1×, and they were incubated for 20 minutes at 37°C, with mechanical homogenization every 5 minutes. Then, PDOs were centrifuged at 1,200 rpm for 5 minutes at 4°C and washed with Hank’s Balanced Salt Solution (Thermo Fisher Scientific). Counting and viability measurements were done using 0.4% Trypan Blue staining solution (Thermo Fisher Scientific) and the Countess 3 Automated Cell Counter (Thermo Fisher Scientific). Expected cells were pelleted again and reseeded in Matrigel.

### 3D PDO drug screenings

To check baseline sensitivity to the drugs and inhibitors used, initial dose-response curves (DRC) were performed with the allosteric inhibitor MK-2206, the nonallosteric one AZD5363 (capivasertib), the ERK inhibitor SCH772984, and oxaliplatin. Initially, PDOs were dissociated into single cells as per the passaging procedure described above, and after automatic counting by the Countess 3 Automated Cell Counter (Thermo Fisher Scientific), 6,000 cells were seeded in 30 µL of Matrigel in 96-well plates. The Matrigel was solidified after a 20-minute incubation at 37°C and 5% CO_2_ and overlaid with 70 µL of complete human organoid media. Twenty-four hours later, the media were removed and replaced with 50 µL of drug-containing media at different concentrations, along with DMSO as a vehicle control, and replenished every 2 days for 3 times. Finally, the drug-containing media was removed and replaced with 10% CellTiter-Blue cell viability assay media (Promega), and after a 3-hour incubation at 37°C and 5% CO_2_, readings were taken in an EnVision plate reader (PerkinElmer). Experiments were conducted in technical and biological replicates. DRCs were represented using GraphPad Prism 9 (RRID: SCR_002798).

### PDO barcoding

Individual cell barcoding was performed using the CloneTracker XP 5M Barcode-3ʹ Library in pScribe4M-RFP-Puro (Cellecta). These expressible barcode libraries enable the tracking and profiling of individual clones because of their integration in genomic DNA (gDNA) and barcode transcription in RNA. To ensure the insertion of a single barcode per cell, lentiviral titration was assessed, and a multiplicity of infection of 0.1 was chosen along with 0.8 μg/mL polybrene, corresponding to 10% infection. One million cells from the organoids were infected following the manufacturer’s protocol. After an overnight incubation in media suspension, cells were pelleted and resuspended in 1 mL of Matrigel in six-well plates, along with 2 mL of media containing 2.5 μg/mL puromycin, as previously calculated by the puromycin killing curve. Following 10 days of puromycin selection, red fluorescent protein–positive cells were checked under the microscope, and they were expanded with normal growth media. After several passages, the parental population (POT) was frozen and characterized. DNA and RNA extractions and libraries sequencing (protocol below) detected approximately 3,500 different barcodes in the final POT.

### Induction of chromosomal instability

F-016 (MSS) and CRC0282 [microsatellite unstable (MSI)] barcoded organoids were treated following the protocol of Bennett and colleagues (15). Briefly, following an overnight treatment with 100 ng/mL of nocodazole (487928, Sigma-Aldrich), the media were removed and replaced with fresh media containing IC_50_ concentrations (15 nmol/L for 3994-117 and 100 nmol/L for CRC0282) of the CENP-E inhibitor GSK923295 (S7090, Selleckchem). After 2 hours, 150 nmol/L of the MPS1 inhibitor BOS172722 (S8911, Selleckchem) was added on top of one well for another 2 hours. Then, all the treatments were removed and replaced with fresh media. Organoids were allowed to grow for 10 days before performing the single-cell sorting and freezing. The experiment was performed in nine replicates, and three replicates were collected at different time points over 15 days to check viability and barcode distribution during the washout phase.

### Generation of drug-resistant organoids

Once the F-016 POT was expanded, 75 million cells were used for the generation of resistant organoids to previously selected inhibitors. Briefly, 2.5 million cells/well were seeded in six-well plates in 1 mL of Matrigel and 2 mL of growth media. Twenty-four hours later, the media were collected and renewed with media containing 2 µmol/L of the selected inhibitors. For the first experiment, MK-2206 and capivasertib were chosen as AKT inhibitors, whereas trametinib, as a MEK inhibitor, and KU-0063794, as an upstream mTOR inhibitor, were also selected. DMSO was always used as the vehicle control.

Following the above-described conditions, individual six-well plates were used for each drug. The bottom three replicas were used for unique treatments, whereas the top three wells were used for a second treatment with drugs involved in different pathways to perform a cross-over screening. For the first experiment, F-016 PDOs were treated for 45 days with the respective first treatment, and then the three bottom wells were harvested and mixed to be used for bulk and single-cell characterization, as well as for barcode amplification from DNA and RNA. The remaining cells were frozen and biobanked in FBS (Thermo Fisher Scientific), containing 10% DMSO (Sigma-Aldrich) for future analysis.

Top three wells were expanded without drug for 2 weeks (drug holidays) and then reseeded under the same conditions as the initial treatment to be treated with the second inhibitor for an additional 45 days. Lately, at the end of the treatment, cells were also expanded without drug for a couple of weeks, characterized, and stored like the initial time points.

As previously described, a total of four inhibitors were selected, and a DMSO control plate was maintained until maximum confluency at day 15. For the cross-over treatment, cells under Akt or mTOR inhibitors were treated with trametinib as a second treatment, whereas in cells initially treated with the MEK inhibitor, Akt or mTOR inhibitors were administered. To check the generation of resistant cells, sensitivity assays by DRCs were performed at the end of each treatment.

For the second experiment, high doses corresponding to the IC_90_ of oxaliplatin and the ERK inhibitor SCH772984 were used in both the MSS and MSI PDOs (Supplementary Table S1), with and without the chromosomal instability (CIN) treatment previously explained, continuously for 5 weeks. Then, the three bottom wells were harvested and used for bulk and single-cell characterization, as well as for barcode amplification from DNA and RNA. The top three wells were expanded without drug for another 3 weeks before harvesting, characterization, and freezing.

### Parental and resistant cell characterization by bulk and single-cell sequencing

Once treatments concluded, organoids were collected and dissociated into single cells using the passaging procedure previously described. Half of the cells were used for bulk analysis, whereas the other half was used for single-cell experiments.

gDNA and RNA were isolated for bulk characterization using the AllPrep DNA/RNA Mini Kit (QIAGEN) according to the manufacturer’s recommendations. It mainly consisted of whole-genome library preparation from 100 ng of gDNA following NEBNext Ultra II FS (New England Biolabs) recommendations, with 20 minutes of enzymatic fragmentation and five PCR cycles. NEBNext Multiplex Oligos for Illumina (96 Unique Dual Index Primer Pairs, New England Biolabs) were used. After pooling, samples were sequenced for low-pass whole-genome sequencing (WGS) with at least 0.1× coverage on a NovaSeq 6000 (Illumina).

With respect to single-cell RNA approaches, a total of 19 single-cell experiments were performed from the parental (POT) and the four inhibitors (MK-2206, capivasertib, trametinib, and KU-63794) after drug removal (under drug) and after expansion (regrowth) for the two lines of treatment. For the second part, 36 single-cell experiments were performed, including the CIN generation and the following treatments with oxaliplatin and the ERK inhibitor SCH772984. After dissociation, single cells were washed with PBS and resuspended in PBS + 0.04% BSA, filtered through a 40 µm Flowmi cell strainer (Sigma-Aldrich), and resuspended at a concentration of 1,000 cells/μL. Viability was confirmed to be >90% in all samples using 0.4% Trypan Blue dye with a Countess 3 Automated Cell Counter (Thermo Fisher Scientific). Those organoids harvested under drug exposure with less than 70% viability were enriched using the Dead Cell Removal Kit (Miltenyi Biotec) according to the manufacturer’s protocol. For an estimation of 5,000 cells, single-cell suspensions were loaded onto a Chromium Next GEM Chip G (10x Genomics) and run in the Chromium Controller to generate single-cell gel beads in emulsions (GEM) using the Chromium Next GEM Single Cell 3ʹ GEM, Library & Gel Bead Kit version 3.1 (10x Genomics). After single-cell RNA sequencing (scRNA-seq) libraries were prepared and the library quality was confirmed with the TapeStation D1000 ScreenTape (Agilent) and a Qubit 3.0 dsDNA HS Assay Kit (Life Technologies), samples were pooled and sequenced on an Illumina NovaSeq 6000 (Illumina) according to the standard 10x Genomics’ protocol.

Single-cell DNA approach was processed following the 10x Genomics Single Cell CNV solution kit (10x Genomics). Briefly, dissociated cells were partitioned in a microfluidic chip to form cell beads and to continue with cell lysis and genomic DNA denaturation. Then, denatured gDNA in the cell bead underwent a second encapsulation with barcoded gel beads to generate GEMs. Following barcoding and amplification, fragments were later pooled to be linked to standard Illumina adaptors. Libraries were finally quantified by D1000 ScreenTape (Agilent) and a Qubit 3.0 dsDNA HS Assay Kit (Life Technologies) and pooled to be sequenced on the NovaSeq S4 chemistry (Illumina) with 100 paired-end reads. Paired-end reads were processed using version 1.0 of the Cell Ranger DNA Pipeline (10x Genomics).

For single-cell multiome sequencing, after organoid dissociation, cells were lysed to obtain single nuclei following the Chromium Nuclei Isolation Kit with RNase Inhibitor (10x Genomics, PN-1000494). Then, using the Chromium Next GEM Single Cell Multiome ATAC + Gene Expression platform (10x Genomics), approximately 5,000 nuclei underwent the transposition step. They were then loaded onto the Chip J Chromatin Controller (10x Genomics) for GEM generation and barcoding. After preamplification, assay for transposase-accessible chromatin (ATAC) and cDNA libraries were independently generated using user recommendations. Libraries were finally pooled and sequenced on the NovaSeq 6000 (Illumina), following specific sequencing read cycles for an output of 25,000 read pairs per nucleus for both conditions.

### Barcode amplification and library preparation

Barcoded organoids at each time point before and after treatment were harvested and pelleted. To study the expressible barcodes, gDNA and RNA were isolated using the AllPrep DNA/RNA Mini Kit (QIAGEN) according to the manufacturer’s recommendations. Although DNA was directly quantified by the Qubit 3.0 dsDNA HS Assay Kit (Life Technologies) and stored for barcode amplification, 300 ng of total RNA was taken for cDNA synthesis with SuperScript II Reverse Transcriptase (Invitrogen). Contrary to a 2D culture, organoids are embedded in Matrigel, and although dead cells remained inside, gDNA is released into the surrounding media. Hence, to track the evolution of each cell lineage during the treatment without replating them, gDNA from apoptotic cells was collected every media change (2 days) to amplify dead cell barcodes, providing us with unparalleled temporal resolution. Collected media were incubated for 2 hours at 55°C with 20 mg/mL proteinase K (Roche). Then, gDNA was purified with 3× SPRIselect beads (Beckman Coulter), followed by an 80% ethanol wash step. DNA was eluted in low Tris-EDTA buffer and quantified using the Qubit 3.0 dsDNA HS Assay Kit (Life Technologies).

Barcode amplification by PCR was performed using 2× ACCUZYME Mix (Bioline) and 10 ng of the extracted DNA/cDNA using the following primers (10 µmol/L):

Forward XP primer: 5ʹ-ACC​GAA​CGC​AAC​GCA​CGC​A-3ʹ
Reverse XP primer: 5ʹ-ACG​ACC​ACG​ACC​GAC​CCG​AAC​CAC​GA-3ʹ

Briefly, 25 μL PCR reactions were performed with an initial denaturation at 98°C for 2 minutes, followed by 35 cycles at 95°C for 15 seconds, 71°C for 5 seconds, 72°C for 10 seconds, and a final extension at 72°C for 3 minutes. Four microliters of the 128-bp PCR product was later checked on a 1.5% agarose gel and quantified for library generation by TapeStation (Agilent). Next-generation sequencing libraries were prepared from 30 ng of the PCR product using the NEBNext Ultra II DNA library preparation kit for Illumina (New England Biolabs) according to the manufacturer’s recommendations and using NEBNext Multiplex Oligos for Illumina (96 Unique Dual Index Primer Pairs, New England Biolabs). Libraries were quantified using the Qubit 3.0 dsDNA HS Assay Kit (Life Technologies) and TapeStation D1000 ScreenTape (Agilent Genomics). Up to 384 samples were pooled for sequencing. Due to unspecific library amplification, 3 ng/μL samples were pooled considering a concentration of 250 to 340 bp in TapeStation. Then, pools were dried to 50 μL using a vacuum concentrator, and electrophoresis in a 2.5% gel was done. A band of 280 bp was cut and purified using a gel purification kit (QIAGEN). Fifty microliters of the eluted product was again quantified by both methods. Next-generation sequencing was performed at the Tumor Profiling Unit of the Institute of Cancer Research using the NovaSeq 6000 (Illumina). A total of 150 paired-end reads and 15% of PhiX were considered, and approximately 5,000 reads per time point were aimed for.

### Phenotypic correlation with barcodes

CloneTracker XP barcode enrichment was performed in single-cell gene expression libraries.

The 10x Chromium 3ʹ Reagent Kit was developed for the amplification of 3ʹ-ends of poly-A+ RNAs. Due to the CloneTracker XP BC14-spacer-BC30 barcode design, located at a fixed ∼150 to 200 nt distance upstream from the poly-A+ site, the transcribed barcodes are captured in the first step of the 10x Chromium 3ʹ protocol.

To effectively read the barcode sequence, an additional PCR amplification step was needed with a barcode-specific primer that is located just upstream of the CloneTracker XP barcode (FBP1). This ensured amplification of the segment of cDNA that contains both the CloneTracker XP barcode and the cell barcode associated with the poly-A+ sequence in the scRNA-seq protocol. In short, after cDNA generation by the 10x Chromium Next GEM Single Cell 3ʹ protocol, 75% of the adaptor-ligated product was used for gene expression libraries, whereas 25% of the amplified cDNA was taken for sequential nested PCR barcode amplification. First, PCR was performed for nine cycles using the following primers:

Partial 10x read 1 primer forward: 5ʹ-ACA​CTC​TTT​CCC​TAC​ACG​ACG​CTC​TTC​CGA​TCT-3ʹ
Specific barcode fragment primer (FBP1) FSeqRNA-BC14-XP Reverse Primer: 5ʹ-GTG​ACT​GGA​GTT​CAG​ACG​TGT​GCT​CTT​CCG​ATC​TCC​GAC​CAC​CGA​ACG​CAA​CGC​ACG​CA-3ʹ

Second round of PCR to increase CloneTracker XP barcoded cDNA sequences used standard 10x p7 and p5 primers for additional six cycles to then proceed to indexing and final library generation. After quality check by TapeStation (Agilent), barcode amplicon libraries were pooled and sequenced. Then, CloneTracker XP barcodes and gene expression were correlated because the 10x barcodes identify cellular gene expression with the clone identifiers.

### Barcode bioinformatics analysis

Barcodes from bulk experiments were quantified using Burrows-Wheeler Aligner (BWA; RRID:SCR_010910; ref. 16) and *FeatureCounts* (RRID:SCR_012919; ref. 17). We first generated a FASTA reference using the full pool of barcodes provided by Cellecta. We then aligned the amplicons using BWA-MEM with custom parameters. The BAM files obtained from BWA were then used to quantify barcode abundances using the *FeatureCounts* implementation of the R package *Rsubread* (18). More specifically, we ran the command *featureCounts*. We filtered reads with mapping quality less than 30 (minMQS = 30). For barcodes enriched from the 10x Chromium library, we used STARsolo (RRID:SCR_021542; ref. 19) with a custom reference index built on the reference FASTA used for BWA.

### Barcode evolutionary modeling

We used the floating barcodes’ abundances to produce an estimate of the fitness value of each barcode. To avoid biases due to the differential efficacy in capturing the DNA across different phases of the experiment, we worked with the relative abundances of each barcode, and to make the calculations tractable for thousands of barcodes, we assumed exponential growth. Under this assumption, if we call $\omega_{i}$ the effective growth rate (the difference between birth rate and death rate) for barcode i, we can write its abundance at time *T* as:

$$N_{i}\left(T\right)=N_{i}\left(0\right)e^{\omega_{i}T}$$

As we were working with relative abundances, it follows that if we assume independence between subclones, the abundance f_i(T) for barcode *i* at time T is simply:

$$f_{i}\left(T\right)=\frac{N_{i}\left(0\right)e^{\omega_{i}T}}{N\left(0\right)e^{\underset{¯}{\omega }T}}=f_{i}\left(0\right)e^{\Delta \omega T},$$

where N(0) is the population size at time 0, $\underset{¯}{\omega }$ is the average growth rate of the population, and Δω is the difference between the barcoded population growth rate and the average population. From this simple model, we derive that the relation between the abundance at sampling time *T* and time *T* − 1 is (after taking the logarithm of both sides):

$$\ln\left(f_{i}\left(T\right)\right)=\ln\left(f_{i}\left(T-1\right)\right)+\Delta T\Delta \omega$$

We then calculate the relative growth rate by fitting the beta coefficients of a linear model on the log abundances using the *fastLM* function of the *RcppEigen* (20) R package.

### scRNA-seq analysis

For the analysis of scRNA-seq data, the fastq files containing the sequential reads were aligned to the reference genome (assembly hg38) using STARsolo version 2.7.9a.

The matrices produced from the alignment were corrected for ambient RNA using the *adjustCounts* function of *SoupX* (21). We then used *DoubletFinder* (22) to estimate the doublet status of each cell. For quality control, filters were applied for the minimum number of genes with nonzero expression and for the percentage of expression derived from mitochondrial transcripts, indicative of dying cells. Specifically, cells with mitochondrial transcripts greater than 30%, fewer than 1,000 expressed genes, and putative doublets from *DoubletFinder* were filtered out.

Data were then analyzed in Seurat (23). Counts were normalized using the *NormalizeData* function. At this point, the 3,000 most variable genes were identified with the FindVariableFeatures routine, which were then scaled (*ScaleData*) and used to compute a principal component analysis (*RunPCA*). The first 25 components, selected based on the amount of variance explained, were used to compute the K-nearest neighbors graph and Uniform Manifold Approximation and Projection (UMAP) with the functions *FindNeighbors* and *RunUMAP*.

We compute gene set scores for the autophagy signature from ref. 24 and the *MSigDB* (25) Hallmark of Cancer MYC Targets version 2 using the function *AddModuleScore*.

### Single-cell ATAC + gene expression analysis

10x Multiomics sequencing data have been processed with Cell Ranger ARC version. We then performed all the quality control and analysis using the R package *ArchR* (26). After generating the arrow files, we used the *filterDoublets* function and filtered out cells with *TSSEnrichment* < 6, number of fragments < 10,000, putative doublets and cells without a valid Unique Molecular Identifiers for both gene expression (GEX) and ATAC libraries. Latent semantic indexes for RNA and ATAC were computed using the *addIterativeLSI* method, using, respectively, the 2,500 most variable genes and the ArchR TileMatrix. Motif annotations for *ChromVAR* (27) were added using the *addMotifAnnotations* function using the *CIS-BP* dataset, and scores were calculated using the *addDeviationsMatrix* method. To compute correlation, we used the *correlateMatrices* function with the Motif Matrix and the Gene Expression Matrix as input. Marker peaks were computed using the *getMarkerFeatures* function, setting the transcription start site enrichment score and the log_10_ (number of fragments) as bias terms. To compute differential transcription factor (TF) motif accessibility, we used the *peakAnnoEnrichment* function.

Background peaks were computed using the *addBgdPeaks* method.

### Deep Archetypal Analysis

Archetypal Analysis (AA) is a dimensionality reduction method that decomposes an input matrix as a convex combination of ideal extreme points called archetypes, and it was first introduced in ref. 28. More in detail, if we assume X∈R to be our N x M input matrix, the classical problem of AA revolves around optimizing the following loss function:

$${\left\|X-ZA\right\|}^{2}\;with\;Z=XB^{T},$$

where *B* and *A* are, respectively, N x K and K x N row stochastic matrices, with *K* being the number of archetypes. We define a matrix *X* to be a row stochastic matrix if it satisfies $x_{ik}\geq 0,\underset{k}{\sum }x_{ik}=1$. The matrix *B* defines the archetype as a convex combination of real data points, whereas the matrix *A* represents the weights of each archetype in reconstructing each cell. This is intuitively equivalent to optimally embedding the data in a convex polytope with *K* vertices (archetypes); it then follows that the archetypes define the convex hull of the polytope.

The original algorithm for finding the minima involves alternating optimization steps in which we first fix *B* and optimize for *A*, and then do the reverse. This solution is generally considered hard to scale as it needs to use the whole dataset for each iteration (29).

To add the possibility of mini-batch learning and to account for possible nonlinear effects in the archetypal composition, we implemented the Deep Archetypal Network presented in ref. 30 into a new tool called MIDAA (31).

In this case, we learn both matrices using a feedforward neural network ${A,\;B\;=\;f}_{\theta }\left(X\right)$. The input is then reconstructed by a specular decoder network $f_{\phi }\left(Z^{*}A\right)=\tilde{X}$. To reduce the complexity of the problem, Z^*^ is fixed and set to a standard simplex with K vertices, and the condition $Z=XB^{T}$ is guaranteed by using an additional term in the loss that becomes:

$${\left\|X-Z^{*}A\right\|}^{2}+{\left\|Z^{*}-BAZ^{*}\right\|}^{2}$$

To fit the scRNA-seq data, we first computed the intersection of the top 2,000 most variable genes in the underdrug phase, the 1,000 most variable in regrowth, and the 500 most variable in the parental sample. We then computed the *Z*-score across all the cells with a valid barcode and used it as input for the AA network. For the single-cell ATAC (scATAC) sequencing from the 10x multiome, given the higher complexity, we used the first 30 components of the latent semantic index as input.

We used the singular value decomposition (which can be interpreted as a generalization of principal component analysis) done on TF-IDF input built by ArchR (26) on a binarized matrix that records for each cell and in windows of 500 bp whether the area is covered by reads or not. Given a binary matrix *X,* the TF-IDF transformation was applied for cell *i* and peak *j*, where the first term (TF) normalizes for the total number of regions in a cell, and the second part (IDF) downweights peaks that are found in many cells (ubiquitous peaks) while upweighting rare but informative peaks.

$$TFIDF_{ij}=\left(\frac{X_{ij}}{\underset{j}{\sum }X_{ij}}\right)\times \log\left(\frac{N}{1+\underset{i}{\sum }X_{ij}}\right)$$

We would mostly expect copy number to influence the coverage of a specific region, that is, a peak in a tetraploid region could look twice as open as one in a diploid region. As our input does not really depend on the coverage of a specific region but only on the fact that it is covered or not, we believe that copy number per se is not a major confounder in our AA on ATAC.

### Copy-number analysis

#### Copy-number analysis from ATAC data

To infer copy-number clones from the ATAC portion of the single-cell multiome, we used CONGAS+ (32). To derive a prior segmentation, we used the inferred copy-number states from the corresponding low-pass DNA analysis, and we did an intersection of the segments among the samples to obtain a unique profile. We then used the function *segments_selector_congas* to filter for putative multimodal segments. We ran the method *fit_congas* and tested a range of clusters from 1 to 15 for 2,000 steps, a learning rate of 0.005, and a temperature parameter for the Gumbel-SoftMax of 10. We chose the best number of clusters that minimized the integrated completed likelihood. The inferred copy-number alteration (CNA) matrix was used as input to MEDICC2 (33) with parameters –*total-copy-numbers* –*input-allele-columns* to build a clonal tree.

#### Copy-number analysis from 10x single-cell DNA data

As per the 10x recommended analysis, CNAs were determined using Cell Ranger (RRID:SCR_017344) with default settings (using GRCh38 as the reference genome). For each sample, a threshold of maximum number of segments per cell was used, as determined by manually assessing 30 random cells from each sample for noisy profiles. The thresholds were 190 for the MSI parental and CENPE-treated organoids, 299 for the CENPE- and MPS1-treated MSI organoid, 291 for the MSS AKT parental organoid, 249 for the CENPE-treated MSS AKT organoid, and 227 for the CENPE- and MPS1-treated organoid. Cells were then binned into 1 Mbp bins, and bins with no overlapping segments in at least one cell were removed. Cells with more than 50% of the genome with a copy number of 0 were removed, as were cells with a mean copy number of more than 20.

#### Copy-number analysis from low-pass WGS

As previously described in ref. 34, FASTQ files were trimmed for adaptor content using Skewer (35) with a minimum length allowed after trimming of 35 bp, keeping only reads with a minimum mean quality of 10 and removing highly degenerative reads (-l 35 -Q 10 -n). Trimmed reads were aligned to hg38 (GRCh38) using BWA-MEM (16). SAM files were sorted and compressed to BAM files, and duplicates were marked using Picard tools (RRID:SCR_006525; https://broadinstitute.github.io/picard/). BAM files were then indexed using Samtools (RRID:SCR_002105; ref. 36).

BAM files were processed using QDNAseq (RRID:SCR_003174; ref. 37) to convert read counts in 500 kb bins across the chromosomes of hg38 into log_2_ ratio data. The 500 kb bins for hg38 were generated according to QDNAseq instructions using the GEM library (38) and normal BAM files from the 1000 Genomes Project (1000 Genomes, ebi.ac.uk, phase III). Data normalization was performed in accordance with the QDNAseq workflow, including sex chromosomes. Bins were required to have a minimum mappability of 65% and 95% non-N bases. The *smoothOutlierBins* function step was removed as it artificially depressed highly amplified bins. The *sqrt* option was used for the *segmentBins* function. Log_2_ ratios in bins and segments were normalized by subtracting the median log_2_ ratio value of all autosome bins.

To call absolute copy number, we used an adapted version of the ASCAT (39) approach, using only log_2_ ratio information and calculating the sum of squared differences between per-bin segmented copy number and the nearest positive integer within a grid search of purity and ploidy parameters, identifying the best-fitting local minima. Based on expected ploidy statuses, we searched for fits in the range of 2.9 to 3.3 for the MSS AKT–mutant organoid samples and 1.9 to 2.3 for the MSI organoid samples, with a minimum purity of 0.98, as these are pure samples. If no local minimum was found, a ploidy of 3.1 was used for MSS AKT–mutant and 2.1 for MSI, with purity being set at 1 for both. Purity and ploidy searches were performed only on the autosome segmented bins, and the X chromosome copy-number status was inferred from the resulting solution.

For CONGAS input (MSS AKT–mutant organoid only), we required segmentation and calling that was comparable across the multiple samples; therefore, we employed multisample segmentation using the copynumber package (*multipcf*, gamma = 10; ref. 40) and called copy number as previously described but across a narrower ploidy range of 3.1 ± 0.1.

All analyses described above were performed blinded, as sequencing samples were anonymized and labeled with coded identifiers not traceable by the bioinformaticians conducting the analysis. Because this was a pilot study using two specific organoids of interest, a formal power calculation for sample size was not required.

### Data availability

Single-cell RNA sequencing data generated in this study are deposited in the European Genome-Phenome Archive at accession number EGAD50000001495. We provided the processed data as annotated Seurat objects and barcode tables in Zenodo (https://zenodo.org/records/11058999). The data analyzed in this study were obtained from The Curated Cancer Cell Atlas at weizmann.ac.il/sites/3CA/colorectal, from The Cancer Genome Atlas using Xena datasets at https://xenabrowser.net/datapages/. Bulk RNA-seq for the 264 metastatic samples was obtained from the Hartwig Medical Foundation (version October 17, 2020; request forms can be found at https://www.hartwigmedicalfoundation.nl/en/data/data-access-request/). All other raw data are available from the corresponding author upon request.

The code to reproduce the analysis and the figures is available on GitHub (https://github.com/sottorivalab/epigenetic_heritability_and_cell_plasticity_reproducibility).

## Results

### Experimental design

**Figure 1.**
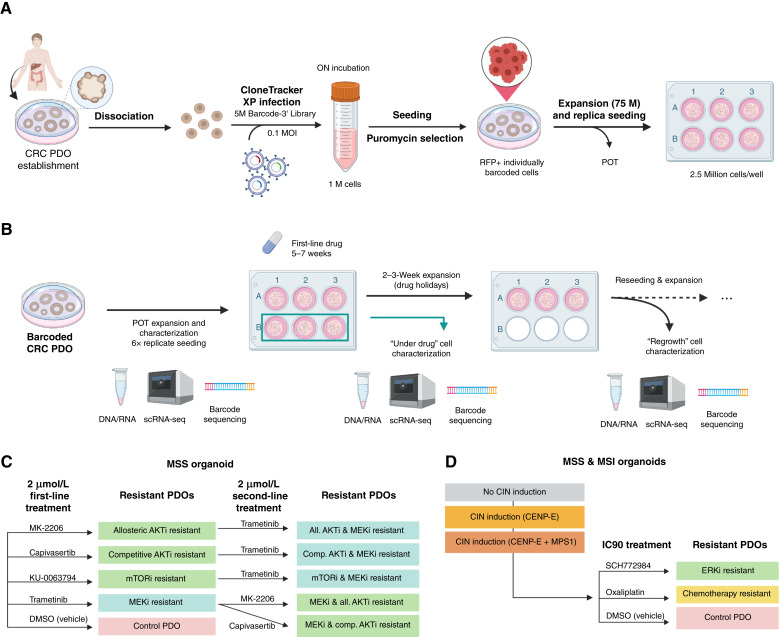
Experimental design of long-term drug resistance evolution in colorectal cancer organoids. **A,** Workflow of lentiviral barcoding in colorectal cancer (CRC) organoid single cells as an evolutionary tracking tool. MOI, multiplicity of infection; RFP, red fluorescent protein. **B,** Experimental design of long-term drug resistance evolution in an MSS AKT–mutant organoid. Bulk DNA profiling was performed for genomic characterization and barcode measurement, as well as scRNA-seq and corresponding single-cell barcode extraction of five “solid” time points over 5 months: parental, under drug 1, regrowth after drug 1, under drug 2, and regrowth after drug 2. Additionally, floating DNA was collected every 2 days from the supernatant to profile barcodes as a “liquid biopsy.” **C,** Cells were exposed to four different sequences of drugs with first- and second-line treatments. **D,** In a second experiment, both organoid lines (MSS and MSI) were exposed to an ERK inhibitor and oxaliplatin. Before drug pressure, CIN was induced with CENP-E inhibitors alone or in combination with the MPS1 inhibitor to assess CIN effects on drug resistance. AKTi, AKT inhibitor; ERKi, ERK inhibitor; MEKi, MEK inhibitor. Created in BioRender. Sottoriva, A. (2025) https://BioRender.com/7toyszr.

MSS colorectal cancers are characterized by large numbers of chromosomal rearrangements, whereas MSI malignancies have largely diploid genomes but a very high number of point mutations due to deficiencies in the mismatch repair machinery. These are the two main genomic subtypes in colorectal cancer. Patient-derived cancer organoids are now an established model system to study cancer biology; they recapitulate a lot of the molecular features of the original patient tumor (41), as well as often their propensity to respond to treatment (14). They better represent human cancers than cell lines while also being more amenable to experimental manipulation than more expensive patient-derived xenografts. In this study, we utilized two PDO lines: first, an MSS AKT–mutant and AKT-amplified organoid that was derived from a metastatic colorectal cancer sample (patient F-016 in ref. 14) and, second, an MSI mismatch repair–deficient (MMRd) organoid that was derived from a primary colorectal cancer (CRC0282 from ref. 13). We infected each organoid with a library of five million random Cellecta Clone Tracker XP expressed barcodes, a form of lentiviral barcoding system that generates poly-A transcripts that can be captured by scRNA-seq (Fig. 1A and Materials and Methods). We ensured that each cell had one barcode by diluting to a 0.1 multiplicity of infection. Single infected cell lineages are subject to high genetic drift due to cell turnover and death, giving rise to an expected final distribution of barcode sizes that scales as a power law (Supplementary Fig. S1). This power law was used as the “null” distribution for comparison against our subsequent evolutionary experiments. We expanded each barcoded population to 75 million cells, and 2.5 million cells/well were plated in multiple six-well plates (Fig. 1A). At baseline, the “parental sample” of the first organoid line (MSS AKT–mutant) had 3,000 to 5,000 individual cell lineages (Supplementary Fig. S1), whereas the second organoid (MMRd) had lower barcode diversity (400 barcodes).

We exposed the MSS AKT–mutant organoid line (see list of driver mutations in Supplementary Table S1) to a set of targeted drugs for 45 days at a high dose of 2 µmol/L concentration (Fig. 1B). We used an allosteric AKT inhibitor (MK-2206), a competitive AKT inhibitor (capivasertib), an mTOR inhibitor (KU-0063794), and an MEK inhibitor (trametinib; Fig. 1C). We applied these drugs sequentially, inducing resistance to drug A, followed by selection pressure from drug B (Fig. 1B). Dose–response curves confirmed that the cells became resistant to those drugs (Supplementary Fig. S2A). We started with six replicas per condition, collected cells after 45 days under the pressure of the drug from three wells, and performed bulk DNA barcode analysis and scRNA-seq. We left the other three wells regrowing in the absence of the drug until confluence and harvested the population again for barcode analysis, bulk DNA profiling, and scRNA-seq. We replated cells into a new six-well plate for a second drug and repeated the profiling as before. Every 2 days during the whole course of the experiment, we collected “floating barcodes” by magnetic bead capture of cell-free DNA released in the media by apoptotic cells: This can be thought of as a “liquid biopsy” of the cell culture. At the end of the experiment, we had five cellular time points directly derived from the system (i.e., a “solid” biopsy) as well as 58 to 61 time points taken from floating barcodes. Floating barcode samples allowed us to characterize the system’s evolution without perturbing it with physical sampling.

In a subsequent experiment, both the MSS and MSI organoids were exposed to a single line of ERK inhibitors (SCH772984) and chemotherapy with oxaliplatin (Fig. 1D) at IC_90_ concentration (Supplementary Table S2; Supplementary Fig. S2B). This enabled the study of chemotherapeutic versus targeted drugs. Furthermore, to probe the contribution of chromosomal changes to drug resistance, we induced CIN in both organoid models by perturbing chromosome segregation (42) using a CENP-E inhibitor, either alone or in combination with an MPS1 inhibitor (Fig. 1D; ref. 15).

**Figure 2.**
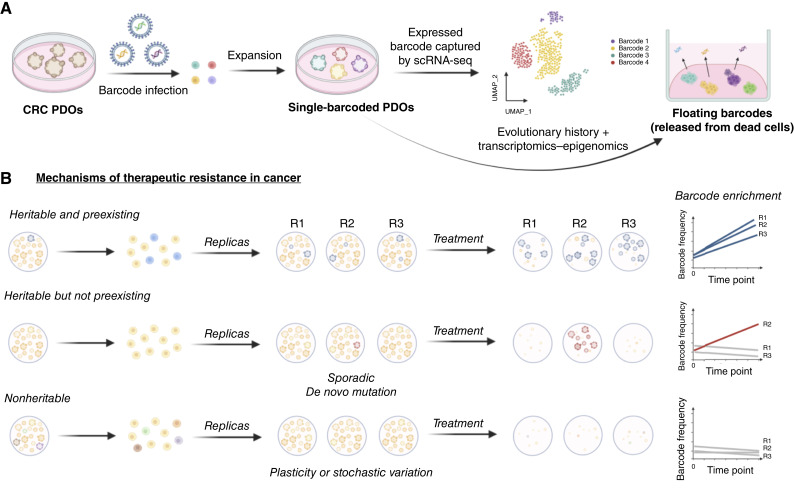
Tracking evolutionary dynamics. **A,** Randomly expressed barcodes allow tracking of cellular evolution matched to single-cell transcriptomics. By collecting the floating barcodes in the supernatant of the culture media, we can also profile the clonal composition of the cells without perturbing them with replating. CRC, colorectal cancer. **B,** Heritable (genetic or epigenetic) preexisting alterations conferring resistance will show as enrichment of the same barcode in different replicas. Heritable *de novo* alterations may also occur during treatment rather than being preexisting; in that case, we expect enrichment for different barcodes between replicas. Finally, nonheritable mechanisms, such as plasticity, will produce drug resistance without any enrichment of barcodes. Created in BioRender. Sottoriva, A. (2025) https://BioRender.com/hu90cn3.

Because the barcodes are expressed, upon scRNA-seq, the full transcriptome of each cell can be matched to the corresponding barcode (Fig. 2A). Single-cell barcodes can be combined with the “floating barcodes” mentioned earlier, collected from the supernatant, to map the evolutionary history of the cell lineage over several time points, along with its gene expression at the end (Fig. 2A). Moreover, barcodes can be used to distinguish between different modes of resistance evolution. If the resistant clone was driven by a heritable alteration (genetic or epigenetic), we expect the same barcodes to emerge in different replicas (Fig. 2B). The emergence of a new alteration driving resistance during exposure to treatment would instead manifest as different barcodes enriched in different replicas. Finally, nonheritable phenotypic plasticity will lead to a resistant phenotype that, however, shows no enrichment in barcodes in any replica (Fig. 2B).

### Resistance evolution to targeted drugs, but not chemotherapy, is heritable, preexisting, and highly repeatable

**Figure 3.**
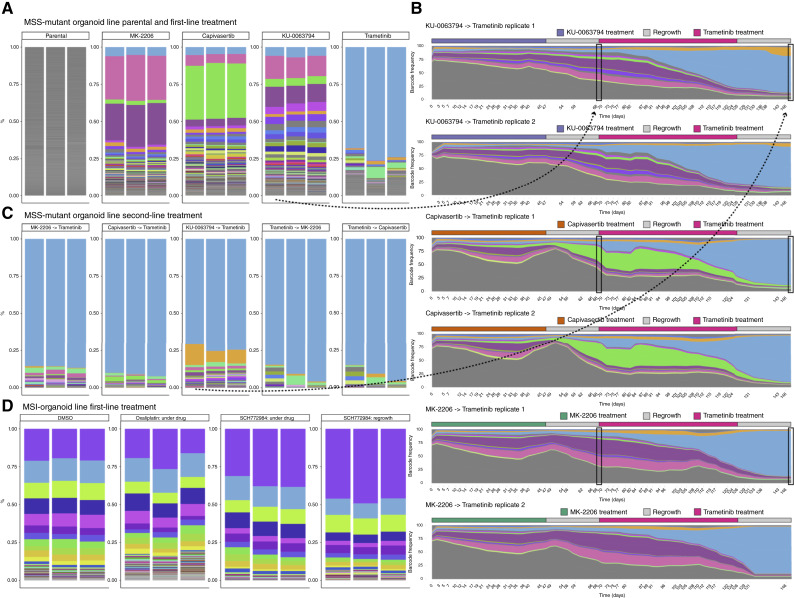
Evolutionary dynamics of barcoded population. **A,** Lentiviral barcode proportions following first-line treatment in MSS AKT–mutant organoid. For each replicate and drug condition, we quantified barcode proportions from genomic DNA, the only exception being trametinib, for which we used the proportion quantified from 10x scRNA-seq. The top 100 barcodes have a unique color across the whole experiment; all the others (<2% abundance) are shown in gray. All the barcodes are quantified after the regrowth period. Selection is evident when compared with the POT. **B,** Reconstruction of clonal dynamics using floating barcodes, extracted from culture media every 2 days over the whole length of the experiment. The dynamics show an evident clonal sweep of the blue barcode after second-line treatment with trametinib. The color code is consistent with **A**. Proportions are smoothed over a rolling average on a window of seven points. **C,** Lentiviral barcode proportions with the second-line treatment in MSS AKT–mutant organoids. **D,** Barcode proportions in MSI organoids after being exposed to chemotherapy (oxaliplatin) and ERK inhibition (SCH772984).

We first focused on the MSS AKT–mutant organoid. In all cases, we found that drugs selected for one or a few subclones that massively expanded during treatment (Fig. 3A). The three biological replicates we seeded per drug showed an almost identical composition of selected barcodes, indicating that those subclones were preexisting in the population and were selected by natural selection. Importantly, as some barcodes traveled together in similar proportions (e.g., pink and purple barcodes in MSS under MK-2206 and KU-0063794, Fig. 3A and B), we cannot exclude that those barcodes belong to the same preexisting subclone (we note that one subclone may have a lot of cells in the parental pool, each of which would be barcoded with a different barcode).

We then examined the floating barcodes to reveal longitudinal clonal dynamics. We confirmed that live cell barcodes at the end of each experiment were highly consistent with floating barcodes, confirming that the latter can be used as a dynamic readout of the live cells at the bottom of the plate (Supplementary Fig. S3A–S3C). Dynamics were extremely similar between replicas, indicating that drug-resistance evolution was highly repeatable under the strong selective pressures of cancer drugs and depended strongly on the initial conditions of the system (Fig. 3B; Supplementary Fig. S4). These data implied that the drug-resistant phenotype was heritable, as the cellular memory encoding the drug-resistant features was passed on to the offspring (i.e., drug resistance was clonally inherited).

Subsequent “second-line” treatment with trametinib (an MEK inhibitor) selected the same subclone as in the first line, which was cross-resistant to the other drugs when selected in the first line (Fig. 3C). We measured the growth rates over time of different clonal subpopulations (barcodes), showing that most surviving clones were not growing under drug pressure but were instead drug tolerant (i.e., barcode frequencies did not increase with time under the drug). When the drug pressure was released, the same subclones massively expanded with a high selective coefficient of up to *s = 0.5* (Supplementary Fig. S5A and S5B). Similarly, in the MSI organoid, treatment with the ERK inhibitor SCH772984 revealed selection for preexisting resistant subclones in a recurrent fashion between replicas (Fig. 3D; Supplementary Figs. S5C and S6; e.g., the purple barcode went from 20% in the parental to 47% in the resistant population). In contrast, chemotherapy with oxaliplatin did not select for any preexisting subclone in either of the two organoids, with the population structure of the cancer remaining unchanged, suggesting that plasticity, instead, could be responsible for resistance (Supplementary Fig. S6). This is consistent with previous findings on the lack of a chemoresistance gene, pointing to plasticity and molecular redundancy as the main mechanisms driving resistance to chemotherapeutics (4, 43).

### Different targeted drugs select for different preexisting clones

We then investigated which subclones (identified by specific barcodes) were selected by different drugs. The two different AKT inhibitors, despite targeting the same signaling mechanism, selected distinct subclones (Fig. 3A). In contrast, the mTOR inhibitor selected similar subclones to the allosteric AKT inhibitor. The MEK inhibitor trametinib was selected for an entirely different subclone that became dominant after drug exposure. The selective advantage of the trametinib-resistant subclone was very strong (Fig. 3A and C; blue clone). The same subclone also proved to be a persister in other drug contexts. For example, during first-line treatment with AKT and mTOR inhibition, the trametinib-resistant subclone did not have a selective advantage but remained at low frequency in the population until the second line of MEK inhibitor was later introduced, which subsequently took over very rapidly.

These data indicate that evolutionary adaptation to targeted drugs was highly repeatable, driven by strong selective pressure on preexisting subclones, and led to the evolutionary divergence of subclones under distinct drugs. Therefore, evolution is determined by the initial conditions of the system (presence/absence of subclones), making it highly predictable, with the opportunity to exploit evolutionary trade-offs for subclones in different drug contexts with specific sequences of drugs. These are the necessary conditions to exploit evolutionary steering strategies (44).

We performed bulk WGS on all MSS organoid samples at regrowth and compared them with the parental sample to identify candidate genetic alterations causing resistance. We found a small set of somatic mutations and CNAs that were different in resistant samples (Supplementary Figs. S7 and S8). However, none of these variants were convincingly linked to previously described candidate mechanisms of drug resistance, with the exception of ABC genes, which encode ATP-binding cassette transporters that play a pivotal role in the development of multidrug resistance in various cancers and tumor progression, including in colorectal cancer (45–47).

### Archetype analysis reveals transcriptional programs

**Figure 4.**
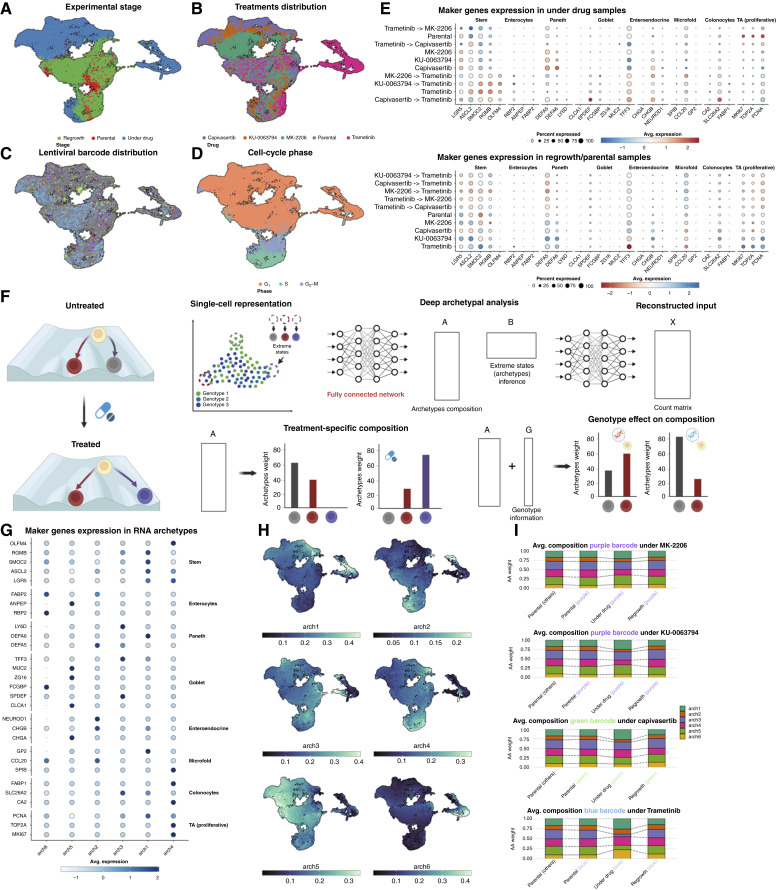
Transcriptional programs show plasticity after drug administration. **A–D, **UMAP of the 37,000 cells in the experiment after quality check filtering, colored, respectively, by experimental stage, drug, barcode, and cell-cycle phase. Cells for which a valid barcode could not be extracted or those with an abundance of less than 1% are shown in gray. Cells in the drug phase tend to strongly cluster by drug, whereas they tend to mix back with the parental cells during regrowth. **E, ***Z*-score distribution of adult colonic cell type markers from ref. 70 shows the presence of distinct differentiation programs inside the organoid. **F,** AA aims at decomposing the input dataset as a convex combination of extreme points by learning two matrices, A and B, which are representative of the archetype weights for each point in the dataset and the matrix that defines the archetypes starting from the input dataset, respectively. Here, we use a deep learning implementation of AA. **G,** We then exploit the weights of matrix A to quantify differences in transcriptional programs across conditions and genotypes. The *Z-*score distribution for the same genes as in **E** is computed by archetype. **H,** Archetype weight distribution over UMAP. **I,** Average archetype weight for different selected barcodes. The trend is consistent with cells going back to the parental phenotype after regrowth. Barcode colors are consistent with Fig. 2.

In MSS organoids, we initially performed scRNA-seq and single-cell barcode extraction (Supplementary Fig. S9A and S9B) from a total of 19 samples (Supplementary Fig. S9C), and we were able to recover the barcodes of 46% of cells on average per sample (30%–64%). Transcriptomes of cells under treatment clustered separately from parental, reflecting the strong influence of the environment on the transcriptomic profile of a cell (Fig. 4A). Cells under different drugs also clustered separately, suggesting each drug influences the transcriptome differently (Fig. 4B). Single-cell barcodes recapitulated barcode distributions from bulk profiling (Fig. 4C). As expected, most cells in S-phase belonged to samples from regrowth after drug pressure (Fig. 4D). We used markers of known cell types in the intestine to classify the transcriptional programs active in each sample (Fig. 4E). The parental sample was characterized by a small but significant component of LGR5+ stem-like cells and a large component of transient amplifying cells. Under the pressure of first-line AKT (capivasertib and MK-2206) and mTOR inhibitors (KU-0063794), which act on the PI3K/AKT pathway, the population maintained a stem cell component but significantly expanded a population with a Paneth signature, as previously reported for patient-derived xenografts under the pressure of EGFR inhibitors (13). Inhibiting the MAPK pathway with the MEK inhibitor (trametinib), even after PI3K/AKT suppression, induced different transcriptional dynamics, in which the LGR5+ stem-like component and partly a Goblet-like component emerged. Conversely, blocking AKT after having previously inhibited MEK gave rise to yet another transcriptional program with a low LGR5+ stem component but high ASCL2+ stem-like cells. At regrowth, stem-like programs were evident, along with a tendency toward microfold cells. For second-line drugs, a wider range of stem-like programs emerge, again together with Paneth cell programs.

Given the heterogeneity of transcriptional programs we found, we hypothesized that a high level of phenotypic plasticity, possibly a reflection of aberrant differentiation pathways, was present in our cell populations. To deconvolve this signal, we developed a new approach based on AA, a dimensionality reduction technique that decomposes an input matrix of gene expression values as a convex combination of ideal extreme gene expression phenotypes called archetypes (28). In this study, we implemented a new Deep AA framework called MIDAA (bioRxiv 2024.04.05.588238v2) to account for nonlinearity in the dimensionality reduction step (Fig. 4F; ref. 30). We found that distinct archetypes were enriched for clearly different cell type markers, highlighting the fact that they represent highly distinct cellular programs, spanning all eight of the cell types we considered (Fig. 4G). Archetype presence clustered distinctively between cells, as expected (Fig. 4H).

To further clarify this and provide a more intuitive representation of the cell-type heterogeneity, we additionally plotted marker expression by Leiden clusters (Supplementary Fig. S10A–S10C) and marker expression on the UMAP (Supplementary Fig. S10D–S10F). This clearly shows regions of cell-type identity/state in the dataset, especially in the cells under the drug. These results indicate that archetypes are robust and can cluster populations of cells in a meaningful, biologically interpretable way. We performed the same analysis in the second line of experiments, obtaining similar results (Supplementary Fig. S11A–S11F). We used archetypes in the rest of the study as a surrogate for cell phenotype or transcriptional program and studied their relationship to clonal evolution (with barcodes), CNAs, and chromatin profiles.

### Generalizability of archetype deconvolution

To further illustrate the power of archetype analysis, we applied the scoring strategy described in ref. 48 to compute an activation score for a set of 14 fundamental biological pathways (Supplementary Fig. 12A; ref. 49) and a set of annotated TFs (Supplementary Fig. 12B; ref. 50). These patterns show how archetypes are quite different in terms of molecular signatures. For instance, archetype 4, prevalent in the resistant blue barcode, has a high PI3K enrichment and overexpression of targets for a set of known TFs involved in proliferative, migratory, or stress‐adaptive phenotypes, such as the ETS family (51), Krüppel‐like factors (52), and the stress regulator XBP1 (53). Having established a molecular foundation for our archetypes, we then investigated the publicly available datasets of scRNA-seq and bulk RNA-seq listed before. We first looked into the scRNA-seq (Supplementary Fig. S13A), exploited the provided cell annotations to isolate the putative cancer cells (11,075 cells), and computed the archetypal contribution using the model trained on our MSS organoid (Fig. 3). We found that the archetype distribution by patient was indeed similar to that in our parental and regrown samples (Supplementary Fig. S13B), with the exception of patient 14, who exhibited an increase in archetype 6. This validates the generalizability of our approach to other colorectal cancer datasets. After validating our model in scRNA-seq, we moved on to bulk RNA, in which we looked at correlation with other molecular and clinical characteristics. A major caveat here is the presence of stroma and normal cells in the sample. We anticipate that some archetype genes might be associated with healthy biological processes, potentially influencing the archetype distribution in bulk RNA-seq. We looked first into The Cancer Genome Atlas (512 colon and rectal cancer samples) and plotted the archetype distribution by sample (Supplementary Fig. S13C). This time, we saw a lot of variability even though average proportions seemed to be consistent with what we found in scRNA-seq. We then exploited the availability of curated nonsynonymous mutational data and information on the copy-number state of each gene computed by GISTIC to score the top correlated and anticorrelated features with our archetypes (Supplementary Fig. S13D and S13E). Although the correlations were not extremely strong, we found some interesting results, such as archetype 6 having a correlation with classical loss in *APC* and *TP53*. On the copy-number variation side, archetype 3 seemed to be correlated with the amplification of *SMAD4*, and archetype 4 seemed to be correlated with the amplification of inflammatory modulators such as NF-κB. We then investigated the association with other data types and found that archetype 3 was significant when stratifying for survival (Supplementary Fig. S13F). Notably, archetype 3 represents a low-proliferative phenotype (as can be seen in the pathway enrichment analysis above and low expression for stem markers in Fig. 3, which was highly enriched in a subset of trametinib under drug cells; Fig. 3B). Finally, we transferred our model to 264 metastatic bulk RNA-seq samples from the Hartwig cohort. Again, we found that although there was quite clear heterogeneity in the cohort, the average value remained concordant with previous observations (Supplementary Fig. S13G).

### The drug environment produces plastic shifts in transcription that are reversible

We examined the composition of clonal barcodes within cells displaying each gene expression archetype. Subclones did not split by archetype; rather, cells from the same barcoded clone gave rise to different archetypes (Fig. 4I), suggesting that “differentiation” into an archetype occurred within each cell lineage. Drug exposure (i.e., the environment) shifted the archetype distribution within a subclone, whereas those distributions were restored to the pretreatment distribution following drug suspension and regrowth (Fig. 4I). Therefore, the selected subclones exhibited transcriptional plasticity, as their archetype distribution within a barcode (which originated from a single cell) initially mirrored the background POT before adapting to the drug exposure and ultimately reverting to the original distribution of states once the drug pressure was removed. We observed a similar pattern in the second experiment, in which we exposed the two organoid lines to ERK inhibitor and chemotherapy, although this was expected under chemotherapy due to the lack of selection for specific barcodes (Supplementary Fig. S11E and S11F). We note that although an overall reversal of the archetype distribution was observed at the whole transcriptome level, some differences in specific genes were noticeable between parental and evolved lines (e.g., see Fig. 4E, bottom).

### The epigenome, but not the transcriptome, of selected subclones reveals their identity

**Figure 5.**
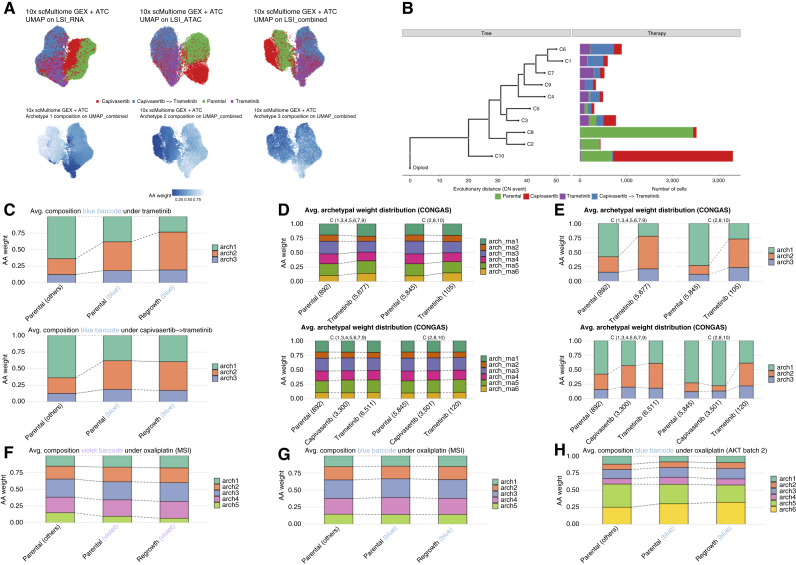
Epigenetic rewiring in resistant populations. **A,** UMAP plots for multiome samples. In the first row, different dimensionality reductions are shown: UMAP done with latent semantic index (LSI) exploiting just RNA information, LSI with just ATAC, and a combined LSI. In the second row, the combined UMAP is colored by archetypal weight. **B,** Clonal tree constructed using CNAs inferred from ATAC data. **C,** Average archetype weights. The blue barcode displays a clear difference in the ATAC profile compared with the others (gray). **D** and **E,** Average ATAC archetype weight for copy-number clones, for RNA archetypes (**D**) and ATAC archetypes (**E**). We split the tree into two major clades: The top one is more abundant in the trametinib samples, and the bottom one is more represented in the parental- and capivasertib-treated samples. The change in archetypal composition is consistent with previous observations from lentiviral lineage tracing. **F** and **G,** Average ATAC archetype weights for the violet and blue barcode in the MSI sample. **H,** Average ATAC archetype weights for the violet barcode in the AKT organoid under oxaliplatin treatment.

We had observed that cells showed phenotypic plasticity upon drug exposure, but drug resistance was nonetheless associated with clonal selection. We hypothesized that the epigenome (in this study measured as chromatin accessibility by ATAC sequencing) could reconcile these apparently contradictory biological observations. We performed single-cell multiome ATAC + gene expression on four samples: parental, trametinib, capivasertib, and capivasertib→trametinib, as those were samples with the strongest bottlenecks. UMAP embeddings, in particular for the scATAC component, clearly separated the cells by treatment with a much stronger signal than the previous scRNA data (Fig. 5A).

We inferred archetypes from the ATAC signal and called three archetypes that were distributed differently across distinct samples. We then used the scATAC signal to infer CNAs with CONGAS (32), identified 10 CNA subclones, and reconstructed their phylogenetic history (Fig. 5B). To assign barcodes to cells, we recovered the lentiviral barcodes from the RNA component of the assay. The efficiency of barcode recovery here was lower [mean per sample = 27% (2%–44%)], and we could only recover a strong signal for the “blue” barcode selected under trametinib and capivasertib. The proportion of epigenetic archetypes, unlike for scRNA, was different in the parental population between the preexisting selected barcode and the rest of the population, suggesting that the memory that induced the selection is encoded in the epigenome (Fig. 5C). The epigenetic memory was maintained in the clone even after expansion under AKT and MEK inhibition (Fig. 5C). To perform the same analysis for other clones with insufficient barcode recovery, we performed subclonal decomposition of the cells based on the copy-number profiles and, again, compared transcriptional programs using the RNA archetypes against their inherited CNA “genotype” (Fig. 5D). The transcriptional programs were very similar when comparing selected versus nonselected subclones, whereas their epigenetic programs were clearly different (Fig. 5E), suggesting that there is a heritable epigenetic memory that encodes many plastic transcriptional phenotypes downstream.

In the context of chemotherapy, both for the MSI organoid (Supplementary Fig. S11E) and the MSS AKT mutant organoid (Supplementary Fig. S11F), the plastic transcriptional rewiring was clear, whereas both the barcodes (clonal structure of the population) and the chromatin remained stable throughout (Fig. 5F–H). This further confirms that plasticity—the potential to exhibit multiple phenotypes—is encoded by a single (epi)genomic configuration.

### Multiomic epigenetic analysis reveals heritable rewiring of the epigenome driving plastic phenotypes

We sought to define the epigenomic configuration that enabled plasticity. First, we looked for recurrent focal changes in chromatin accessibility in promoters and enhancers between pre- and posttreatment cells. We found a very small set of differentially accessible peaks in the promoters of differentially expressed genes (Supplementary Fig. S14), of which, only one was of particular interest: increased accessibility to the promoter of *SETBP1*, a known epigenetic regulatory hub (Supplementary Fig. S15A; ref. 54). We then also looked at genome-wide epigenomic rewiring, focusing on accessibility at TF binding sites across the genome.

A significant correlation emerged between TF gene expression levels and changes in accessibility at their respective binding sites, identifying a subset of TFs with enriched motifs, involving WNT and MAPK signaling pathways, as well as homeobox domains (Supplementary Fig. S15B–S15E). Motif enrichment was then mapped into the UMAP, showing localization of chromatin accessibility patterns (Supplementary Fig. S15F). A previous study in pancreatic cancer showed that resistance to trametinib was associated with increased autophagy and reduced MYC activity (24). Similarly, we observed the autophagy expression signature under trametinib treatment (Supplementary Fig. S15E). However, barcode analysis showed that the signal was not derived from the fully resistant subclone (the “blue” trametinib-resistant barcode) but from the residual persister cells that would eventually die after being overtaken by the “blue” barcode (Supplementary Fig. S15G and S15H).

In this study, we started by asking the following question: “If a barcoded clone X is selected by a drug from the original POT, what made it different from the rest?” We found that at the transcriptome level, the selected clone was initially indistinguishable from the rest of the cells, so why was it selected? We then looked at the genetics, but again nothing stood out. However, when we analyzed the chromatin profile, we found that the selected clones exhibited epigenetic differences from the rest of the POT. Such differences remained during the drug treatment and at relapse, even when the transcriptome was rewiring back to the original state. Hence, our findings suggest that it is the combined evolution of the barcodes, chromatin, and transcriptomes over time and space that provides evidence of what is heritable and what is plastic, as per the design of the experiment.

### Induced CIN alters evolution but not phenotype

**Figure 6.**
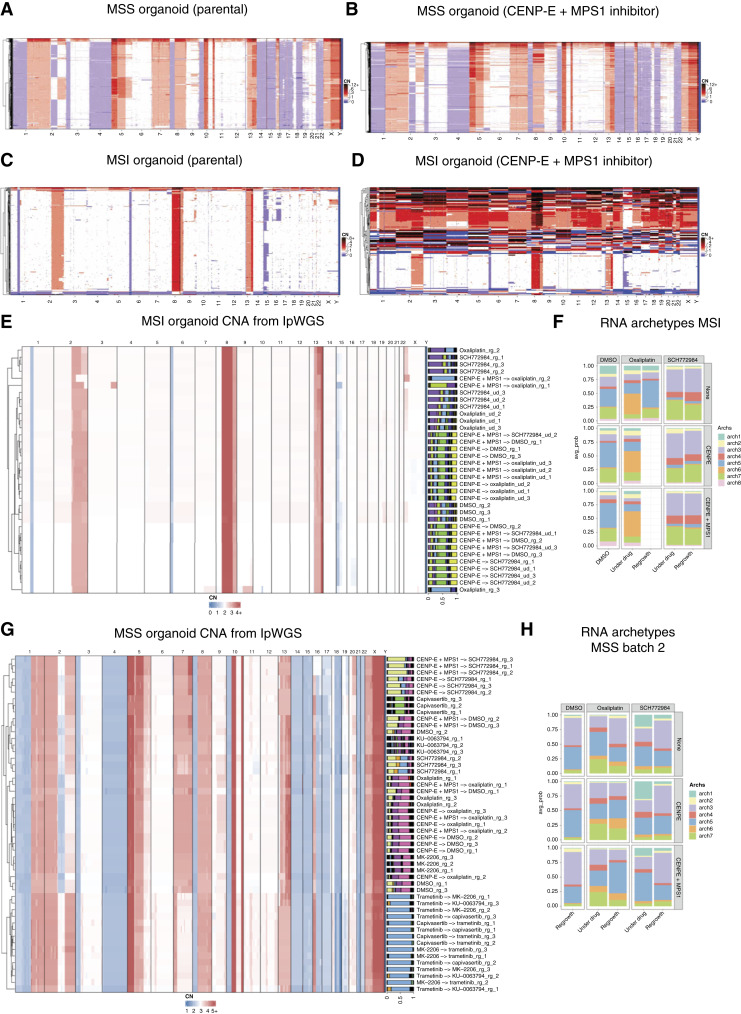
Inducing CIN before drug pressure. **A** and **B,** Single-cell copy-number profiles for the MSS AKT–mutant organoid untreated and after treatment with CENP-E and MPS1 inhibitors. This organoid seems to be stable and resilient to drug-induced alterations. **C** and **D,** Same as **A** and **B** but for the MSI organoid. In this case, the situation is different, and CENP-E and MPS1 inhibition causes a massive increase in instability, with a residual bulk of the population similar to the parental one. **E** and **F,** Bulk copy-number profile and population structure as recapitulated by barcode composition. Barcode composition matches copy-number clones, particularly evident with the blue trametinib-resistant barcode in the MSS AKT–mutant organoid. It is also clear how CENP-E inhibition induces significant changes in the population structure in the MSI organoid. **G** and **H,** RNA archetypes distribution for MSI and MSS organoids. As expected, drug exposure induces a specific transcriptional phenotype, but the induced instability acts only on the population structure, with minimal influence on the transcriptome. lpWGS, low-pass WGS.

CIN correlates with tumor progression (34, 42, 55) and is likely to be involved in drug resistance as well. In recent work, we showed that chromosomal configurations, although grossly altered, were relatively stable across long periods and through treatment in patients with colorectal cancer (bioRxiv 2020.03.26.007138). Immune predation has also been shown to remove chromosomal aberrations (56). The data suggested that negative selection was stabilizing the karyotypes and hence that cancers were sitting on a fitness peak in which all additional chromosomal variation was largely neutral, constrained around a fitness maximum. We had two questions: Can we push cancers away from that maximum by inducing additional CIN? Would changing the initial chromosomal configuration in this way alter the future evolution of drug resistance? We induced CIN with CENP-E and MPS1 inhibitors (see Materials and Methods) in both organoid lines. The MSS organoid was MMRp and chromosomally highly altered to start with (Fig. 6A), and exposure to the agents above proved relatively toxic (IC_50_ = 15 nmol/L for the MSS and 100 nmol/L for MSI organoid lines). In the MSS line, this caused chromosomal rearrangements that nevertheless disappeared with time, and the cellular population returned to the chromosomal configuration of the parental line (Fig. 6B). We found no alterations to the evolution of the tumor following exposure to drugs (Supplementary Fig. S4). The MMRd organoid, instead, was chromosomally stable and mostly diploid (Fig. 6C). It was much more tolerant to CIN-inducing agents, and massive rearrangements in the chromosomal configuration were produced (Fig. 6D) that had profound repercussions on future evolution. The population structure, as reflected by barcode composition (Fig. 6E and F), completely changed, and so did further evolution to targeted agents (Supplementary Fig. S4). However, the transcriptional profiles driving resistance were not different from non-CIN–induced samples (Fig. 6G and H). This highlighted the different selective pressures acting on the cancer and convergent evolution for drug resistance phenotypes, which may be independent of DNA alterations.

### A one-to-many (epi)genotype-to-phenotype mapping drives resistance

**Figure 7.**
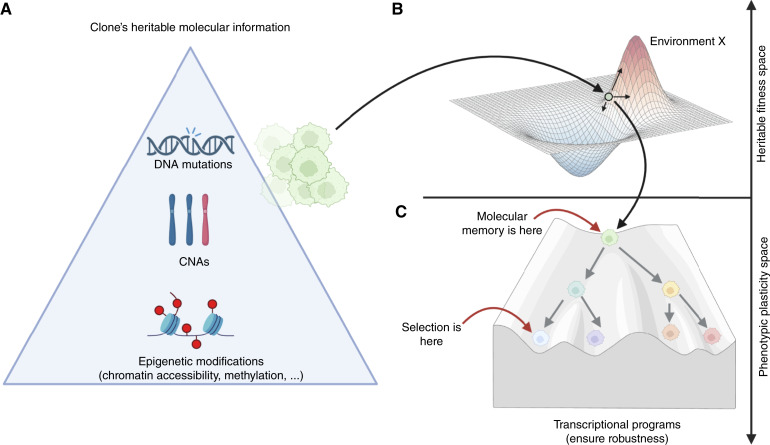
Heritability and plasticity of cellular phenotypes. **A **and** B,** We propose a conceptual model in which genetic mutations and CNAs, together with heritable chromatin accessibility profiles, determine the cellular memory of a certain clone (**A**), positioning it within a certain heritable fitness landscape (**B**). However, the clone does not manifest as a single transcriptional phenotype but rather as a set of transcriptional programs that could be represented within a Waddington landscape, similarly to those that regulate development (**C**). Darwinian selection acts at the phenotypic level, likely exerting selective pressure at the base of the Waddington landscape. This pressure may favor only a subset of a clone’s transcriptional programs, whereas the molecular memory encoding these programs may also retain other plastic phenotypes as a side effect. This may explain the persistent phenotypic heterogeneity and plasticity of cancer clones despite the strong selective pressure of treatments that, instead, should select for a single fittest phenotype. Created in BioRender. Sottoriva, A. (2025) https://BioRender.com/p3al29z.

Our evolutionary analysis demonstrates clear Darwinian dynamics driving resistance. At the same time, the transcriptional programs do not show such a preexisting memory at the RNA level. Given the lack of genetic alterations directly responsible for resistance, one could speculate on a possible conceptual model in which the combination of genetic alterations, such as SNVs and copy numbers, and also, importantly, the epigenetic configuration (Fig. 7A) determines in what specific location on the fitness landscape a subclone resides (Fig. 7B). Each subclone follows a one-to-many mapping, in which its cellular memory, encoded in its genome and epigenome, defines the range of transcriptional phenotypes it can express under different environmental conditions. Therefore, although a cell retains a long-term memory of phenotypic potential, the specific phenotype displayed can shift in response to environmental selective pressures. This potential for transcriptional heterogeneity enables rapid adaptation to changing microenvironments. Darwinian selection acts on clone “memory”—the (epi)genomic configuration that preserves a cell’s ability to access adaptive phenotypic states under drug selective pressure (Fig. 7C). We stress that the speculative model in Fig. 7 will require additional experimentation to be validated; this is the focus of further studies in the context of patient-derived experimental evolution.

## Discussion

Despite significant advances in cancer therapy and molecular characterization, only a limited subset of genetic alterations driving drug resistance has been identified. Typical examples include gatekeeper mutations conferring resistance to targeted therapies, such as in lung cancer (57); mutations in signaling pathways downstream of the targeted receptor (e.g., RAS/RAF mutations; ref. 58); and genetic alterations that revert a drug-induced vulnerability (59). In endocrine therapy for breast and prostate cancers, resistance-associated mutations in ER (60) and AR (61) have been documented. However, in most cases, these mechanisms are not dominant within the tumor, supporting a model of polyclonal drug resistance (62). Additionally, although chemotherapy remains the backbone of treatment for most malignancies, recurrent genetic alterations conferring chemotherapy resistance are not well documented. This lack of insight presents a major obstacle to the development of more effective treatments.

To address this gap, we leveraged PDOs, a model system increasingly used in translational cancer research (41). Unlike traditional cell lines, which often harbor artificial chromosomal changes and exhibit profoundly altered epigenetic landscapes, PDOs retain key features of the original tumor, including genomic stability, epigenetic memory, differentiation capacity, and tumor heterogeneity. These attributes make them a more physiologically relevant system for studying drug resistance mechanisms in a controlled yet clinically meaningful setting. By exploiting this system, we designed evolutionary experiments to dissect the complex nature of drug resistance, distinguishing heritable genetic and epigenetic evolution from transient phenotypic plasticity.

Using cellular barcoding and lineage tracing, we found that under targeted drug pressure, the preexisting heritable trait of plasticity is selected in a highly repeatable and predictable manner. Resistance to targeted therapies arises predominantly through the selection of preexisting subclones characterized by stable chromatin accessibility states, which facilitate robust transcriptional plasticity (63). This supports a model in which epigenetic memory is a key determinant of resistance evolution. In contrast, chemotherapy resistance seems to emerge via transient phenotypic plasticity rather than stable clonal selection, highlighting distinct evolutionary trajectories between targeted therapies and cytotoxic agents (64). Applying evolutionary theory to barcode dynamics over time, combined with machine learning–based single-cell transcriptomics, we identified a fundamental paradigm in cancer resistance: The (epi)genotype–phenotype map is inherently complex but governed by a genetic–epigenetic memory that enables the expression of multiple phenotypic states (65). Which phenotype is manifested depends on environmental conditions, such as drug exposure, potentially explaining the high level of phenotypic plasticity observed in tumors despite strong selection pressures. This also accounts for clonal expansions lacking a clear genetic driver, suggesting that epigenetic heritable traits play a role in resistance.

Our findings carry important implications for therapeutic strategies. As resistance to targeted therapies is driven by preexisting epigenetic memory rather than solely by genetic mutations, treatment approaches must extend beyond genetic biomarkers to counteract epigenetic plasticity (65, 66). Combining targeted inhibitors with chromatin-modifying drugs may limit the expansion of preresistant subclones, whereas intermittent or adaptive dosing strategies could exploit evolutionary trade-offs to prevent the emergence of highly plastic, drug-resistant populations (67). For chemotherapy, interventions targeting transient adaptive states or exploiting dynamic vulnerabilities during phenotypic transitions may prove more effective. Furthermore, integrating chromatin accessibility profiling with genomic analysis could serve as a predictive biomarker approach to identify tumors prone to epigenetically mediated resistance.

Although the use of PDOs presents unique advantages (41), it is crucial to acknowledge their limitations. Unlike cell lines, which are widely accessible and easily manipulated, organoids require more complex conditions (68). Additionally, although PDOs maintain key features of primary tumors, their ability to fully recapitulate the *in vivo* tumor microenvironment is limited (68). By situating our work within the broader landscape of cellular barcoding applications, we emphasize that our approach, while reductionistic, offers valuable insights into the evolutionary dynamics of drug resistance. However, our study focused on two representative colorectal cancer models, selected based on their sensitivity to drugs that induce cell death rather than cytostatic or slowed growth. Although these models allowed for high-resolution evolutionary analysis, they may not capture the full heterogeneity of resistance mechanisms across different tumor subtypes. Expanding this research to a broader range of patient-derived models will be essential for generalizing our findings.

Moreover, although cellular barcoding provides a powerful tool for tracking clonal evolution, translating these findings to *in vivo* tumor ecosystems requires further validation. In model systems, barcoding enables high-resolution deconvolution of phylogenetically related subclones and their transcriptomic states. However, new single-cell DNA sequencing technologies will be necessary to approximate this level of resolution in patient samples, allowing us to validate our observations in a clinical context (69).

Our study provides a mechanistic understanding of cellular plasticity within the clonal evolutionary framework and establishes a one-to-many (epi)genotype-to-phenotype paradigm in cancer resistance. These insights have profound translational implications for colorectal cancer treatment, reinforcing the need for therapeutic strategies that target both genetic and epigenetic determinants of resistance. Future work should focus on expanding these analyses to a broader set of patient-derived models to better capture tumor heterogeneity. Additionally, leveraging single-cell multiomics and chromatin accessibility profiling may help identify predictive biomarkers of resistance and guide personalized treatment strategies. By integrating evolutionary principles into therapeutic design, we may be able to develop more effective strategies to counteract drug resistance and improve patient outcomes in colorectal cancer.

## Supplementary Material

Supplementary Table 1 List of driver mutations on MSS AKT-mutant organoid line after exposure to a set of targeted drugs

Supplementary Table 2 IC90 values assessed by CellTiter-Blue cell viability assay

Supplementary Figure 1 Distribution of lentiviral barcodes from the POT across biological and technical replicates

Supplementary Figure 2 Drug response curves of the resistant organoids

Supplementary Figure 3 Cell barcodes vs floating barcodes

Supplementary Figure 4 Floating barcodes evolution for the whole cohort

Supplementary Figure 5 Relative fitness distribution computed over floating barcodes abundance

Supplementary Figure 6 Lentiviral barcodes proportion

Supplementary Figure 7 Allele frequency distribution in parental vs capivasertib->trametinib treated organoids

Supplementary Figure 8 Copy number profiles of treated and untreated organoids from low-pass WGS

Supplementary Figure 9 Lentiviral barcodes distribution in 10X scRNA-seq

Supplementary Figure 10 Normalised expression for a set of marker genes for colon cell and UMAP feature plots

Supplementary Figure 11 Transcriptional Landscape of organoids treated with Oxaliplatin and SCH77298

Supplementary Figure 12 Normalised Progeny pathway and transcriptional factor scores by archetypes

Supplementary Figure 13 Cell type and Archetype composition in TCGA Colorectal Cancer samples

Supplementary Figure 14 Trackplot of peaks enriched in gene promoters

Supplementary Figure 15 Biological characterisation of trametinib resistant population
