# Supplementary material for: Epigenetic Heritability of Cell Plasticity Drives Cancer Drug Resistance through a One-to-Many Genotype-to-Phenotype Paradigm
Source: Cancer Res. 2025 Jun 11;85(15):2921–38. doi: 10.1158/0008-5472.CAN-25-0999 (PMC12314525; doi:10.1158/0008-5472.CAN-25-0999)
Supplement: Supplementary Table 2 — IC90 values assessed by CellTiter-Blue cell viability assay [file can-25-0999_supplementary_table_2_suppst2.docx]

| **IC_90_ values** | **Oxaliplatin (μM)** | **SCH772984 (μM)** |
| --- | --- | --- |
| MSI Parental | 25 | 0.5 |
| MSI CENPEi | 25 | 1 |
| MSI CENPEi + MPS1i | 50 | 1 |
| MSS AKT Parental | 50 | 1 |
| MSS AKT CENPEi | 50 | 1 |
| MSS AKT CENPEi + MPS1i | 50 | 1 |

**Supplementary Table 2. IC_90_ values assessed by CellTiter-Blue cell viability assay**

Organoids were treated with serial dilutions of the drugs along with DMSO as a vehicle control, and treatment was replenished every two days for three times. Experiments were conducted in technical and biological replicates. Plates were assessed by CellTiter-Blue cell viability assay media (Promega).
