# Supplementary material for: Epigenetic Heritability of Cell Plasticity Drives Cancer Drug Resistance through a One-to-Many Genotype-to-Phenotype Paradigm
Source: Cancer Res. 2025 Jun 11;85(15):2921–38. doi: 10.1158/0008-5472.CAN-25-0999 (PMC12314525; doi:10.1158/0008-5472.CAN-25-0999)
Supplement: Supplementary Figure 1 — Distribution of lentiviral barcodes from the POT across biological and technical replicates [file can-25-0999_supplementary_figure_1_suppsf1.pdf]

Supplementary Figure 1

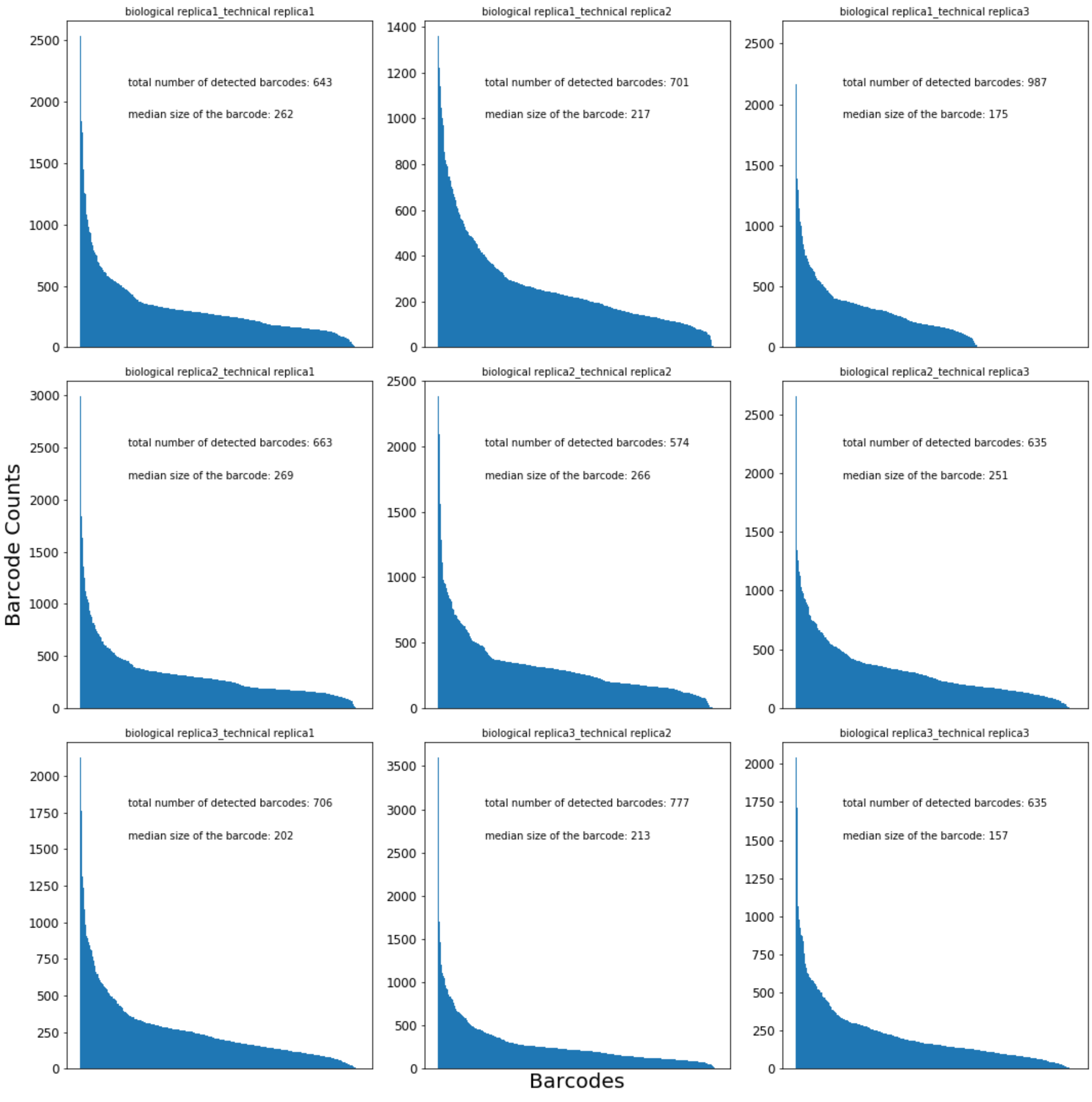

Supplementary Figure 1. Distribution of lentiviral barcodes from the POT across biological and technical replicates. The plot shows how lentiviral barcode proportions in absence of selective pressure follow and expected power-law distribution and how the number of barcodes retrieved is consistent across replicates.
