# Supplementary material for: Epigenetic Heritability of Cell Plasticity Drives Cancer Drug Resistance through a One-to-Many Genotype-to-Phenotype Paradigm
Source: Cancer Res. 2025 Jun 11;85(15):2921–38. doi: 10.1158/0008-5472.CAN-25-0999 (PMC12314525; doi:10.1158/0008-5472.CAN-25-0999)
Supplement: Supplementary Figure 2 — Drug response curves of the resistant organoids [file can-25-0999_supplementary_figure_2_suppsf2.pdf]

Supplementary Figure 2

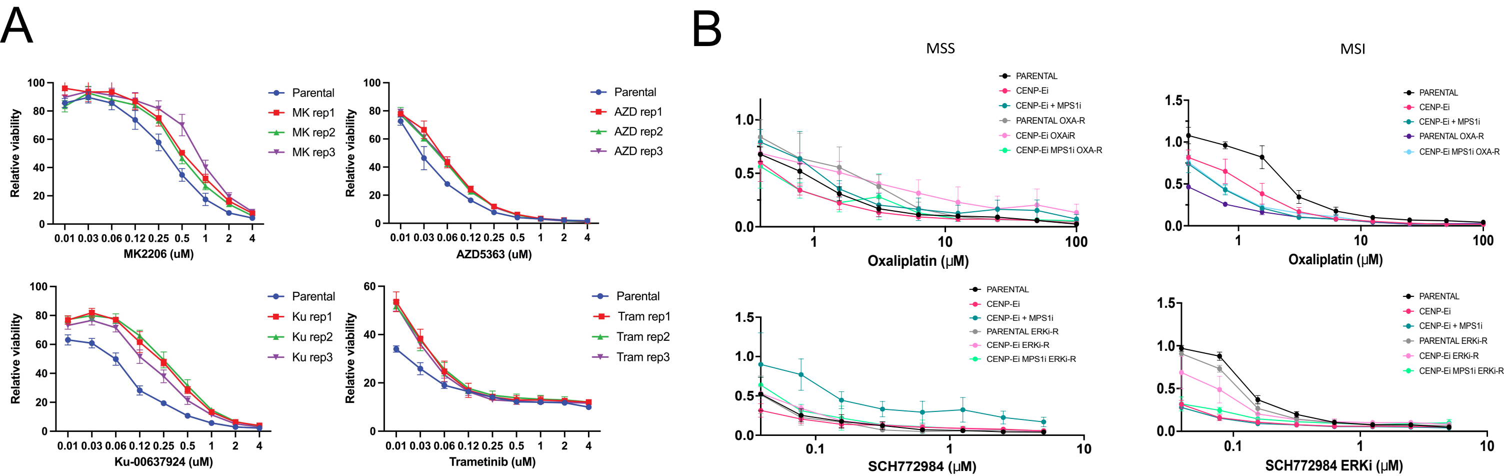

Supplementary Figure 2. Drug response curves of the resistant organoids. (A) Drug response curves related to the first experiment using the MSS AKT organoids after the long-term treatments with the 4 targeted inhibitors (MK-2206, capivasertib, trametinib, and KU-0063794) during the recovering stage showing they remained resistant after the drug removal in all replicates. (B) Drug response curves related to the second experiment after the CIN treatment and long-term exposure to oxaliplatin and SCH772984 after the 3 weeks of recovery without the drug, showing that the cells recovered sensitivity after the heavy treatment.
