# Supplementary material for: Epigenetic Heritability of Cell Plasticity Drives Cancer Drug Resistance through a One-to-Many Genotype-to-Phenotype Paradigm
Source: Cancer Res. 2025 Jun 11;85(15):2921–38. doi: 10.1158/0008-5472.CAN-25-0999 (PMC12314525; doi:10.1158/0008-5472.CAN-25-0999)
Supplement: Supplementary Figure 3 — Cell barcodes vs floating barcodes [file can-25-0999_supplementary_figure_3_suppsf3.pdf]

Supplementary Figure 3

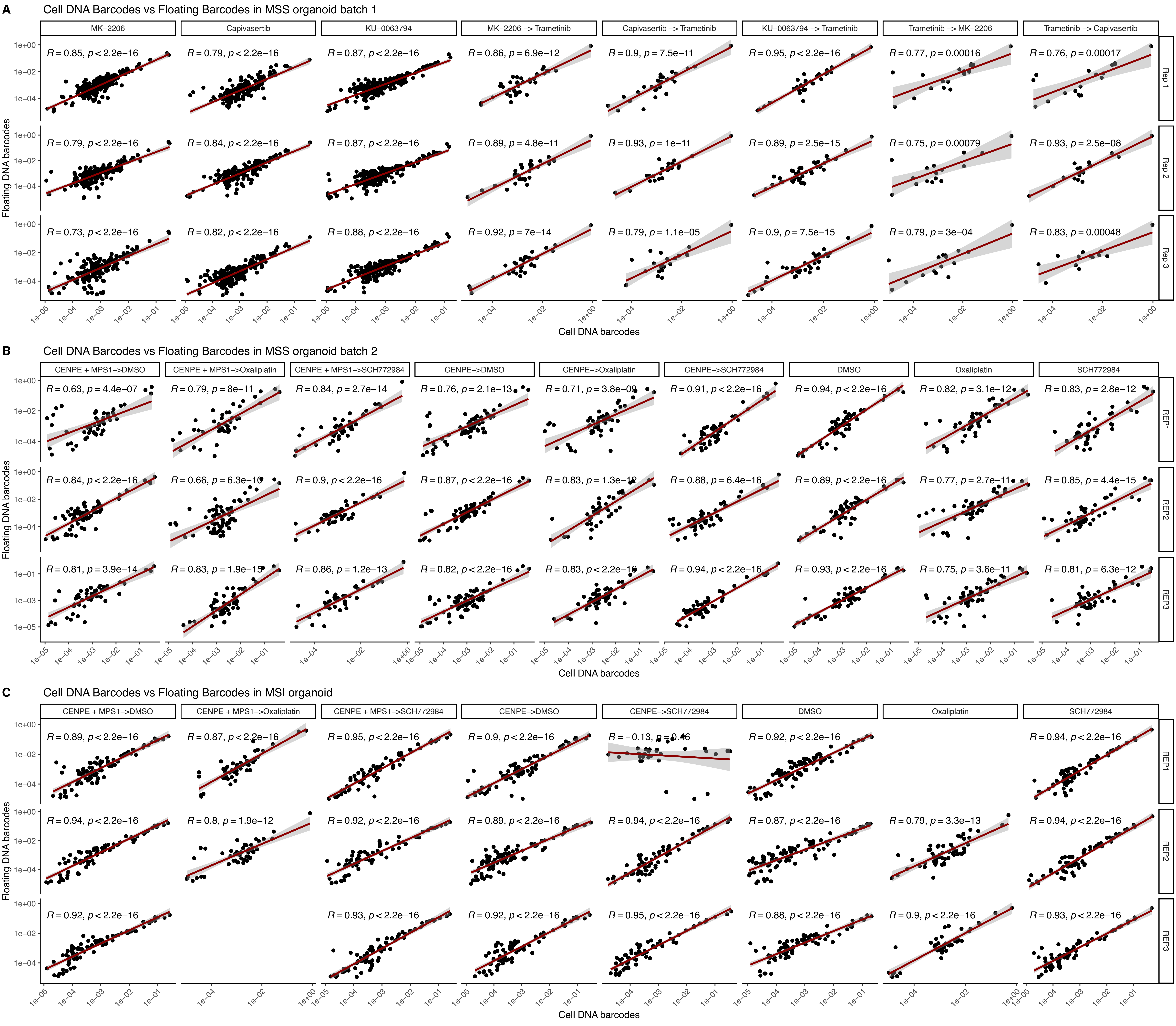

Supplementary Figure 3. Cell barcodes vs floating barcodes. Correlations between cell barcodes and floating barcodes collected at the same timepoint from the supernatant. (A) in the MSS organoid batch 1; (B) In the MSS organoids batch 2; (C) In the MSI organoid.
