# Supplementary material for: Epigenetic Heritability of Cell Plasticity Drives Cancer Drug Resistance through a One-to-Many Genotype-to-Phenotype Paradigm
Source: Cancer Res. 2025 Jun 11;85(15):2921–38. doi: 10.1158/0008-5472.CAN-25-0999 (PMC12314525; doi:10.1158/0008-5472.CAN-25-0999)
Supplement: Supplementary Figure 4 — Floating barcodes evolution for the whole cohort [file can-25-0999_supplementary_figure_4_suppsf4.pdf]

Supplementary Figure 4

Full figure at https://figshare.com/articles/figure/Supplementary_Figure_4/29040722?file=54461468

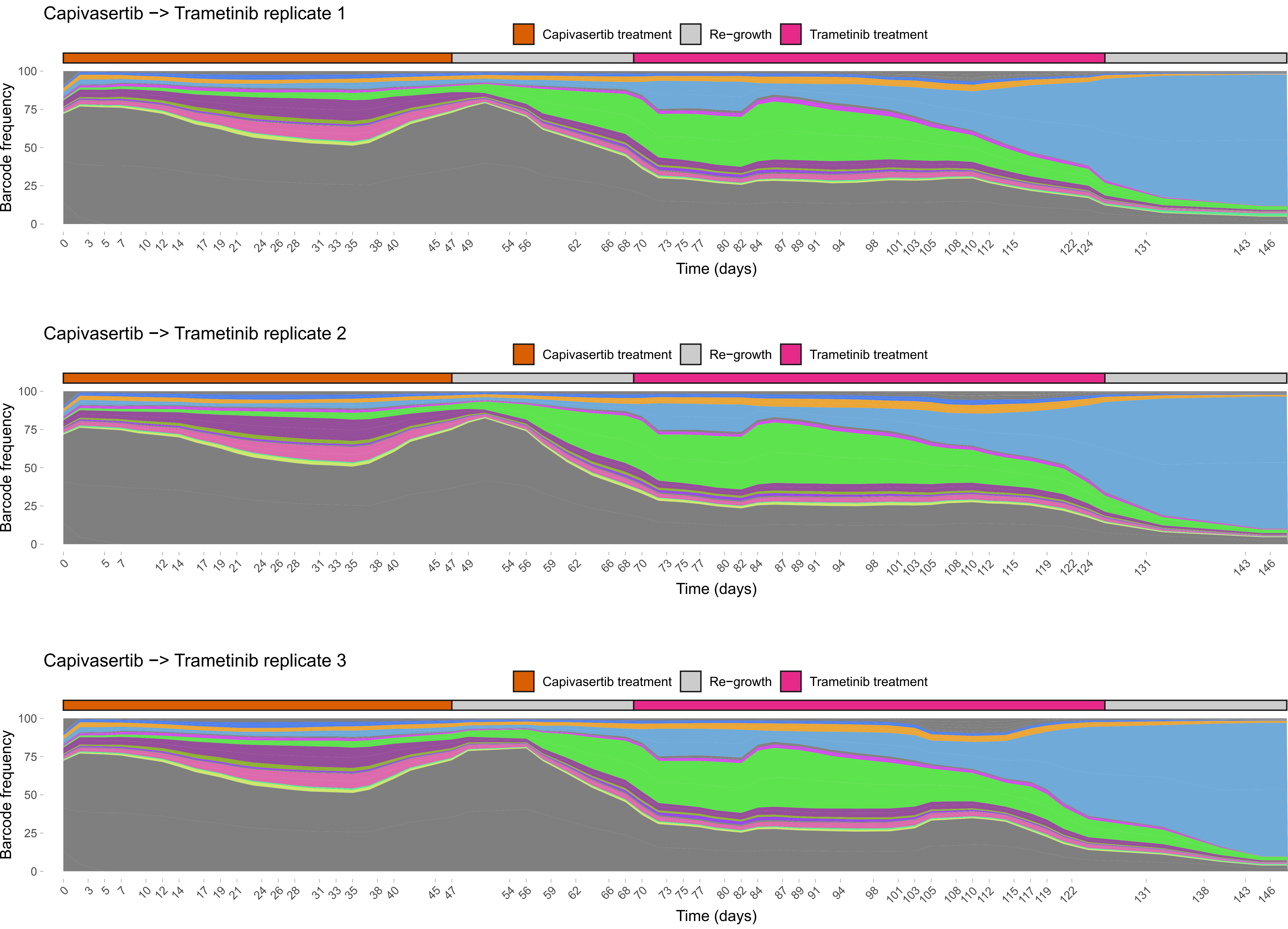

Supplementary Figure 4. Floating barcodes evolution for the whole cohort. Fishplots as in Figure 2 panel B showing the clonal dynamics of all the replicates and the sample in the experiment.
