# Supplementary material for: Epigenetic Heritability of Cell Plasticity Drives Cancer Drug Resistance through a One-to-Many Genotype-to-Phenotype Paradigm
Source: Cancer Res. 2025 Jun 11;85(15):2921–38. doi: 10.1158/0008-5472.CAN-25-0999 (PMC12314525; doi:10.1158/0008-5472.CAN-25-0999)
Supplement: Supplementary Figure 5 — Relative fitness distribution computed over floating barcodes abundance [file can-25-0999_supplementary_figure_5_suppsf5.pdf]

Supplementary Figure 5

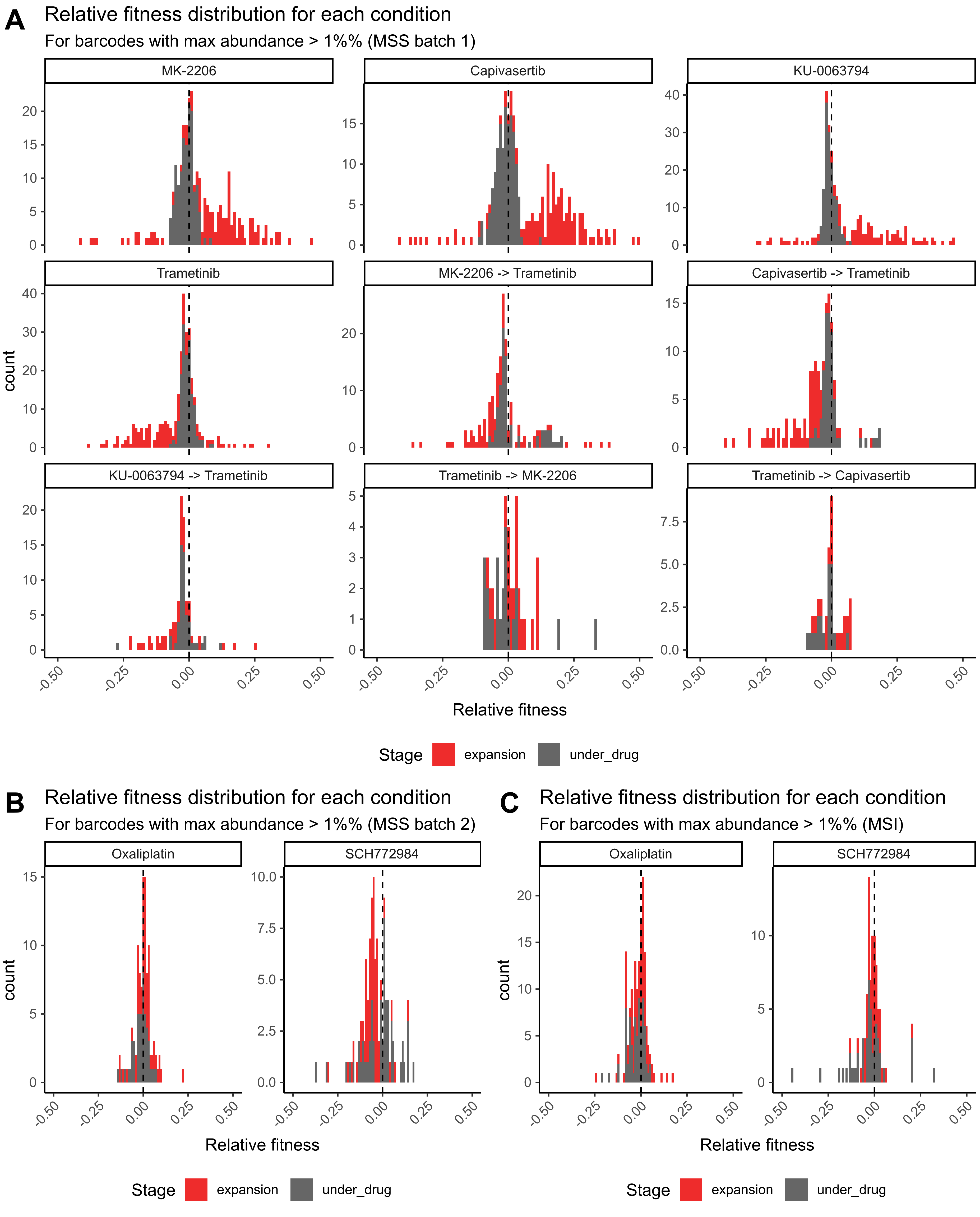

Supplementary Figure 5. Relative fitness distribution computed over floating barcodes abundance. Relative fitness distribution shows high variability in re-growth phase during first-line treatment. Coefficients were calculated by assuming exponential growth and independence between different clonal populations. (A) MSS AKT organoid first batch, (B) MSS AKT organoid second batch, (C) MSI organoid.
