# Supplementary material for: Epigenetic Heritability of Cell Plasticity Drives Cancer Drug Resistance through a One-to-Many Genotype-to-Phenotype Paradigm
Source: Cancer Res. 2025 Jun 11;85(15):2921–38. doi: 10.1158/0008-5472.CAN-25-0999 (PMC12314525; doi:10.1158/0008-5472.CAN-25-0999)
Supplement: Supplementary Figure 6 — Lentiviral barcodes proportion [file can-25-0999_supplementary_figure_6_suppsf6.pdf]

Supplementary Figure 6

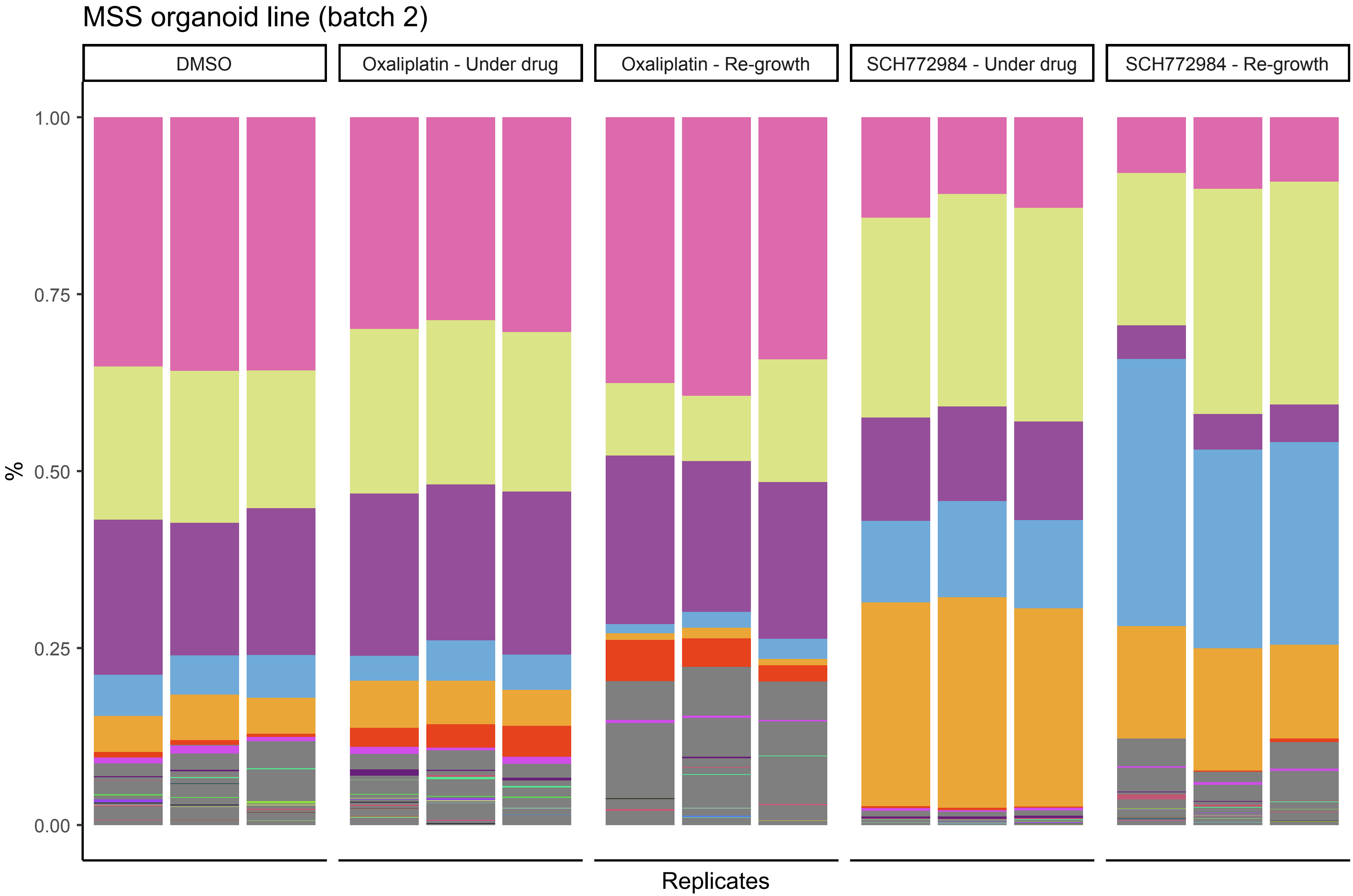

Supplementary Figure 6. Lentiviral barcodes proportion for the AKT organoid after the CENP-E/MPS1 perturbation experiment and during the resistant generation with oxaliplatin and SCH772984. Colours are consistent with Figure 2 panel A and B, so that grey barcodes are lowly abundant barcodes are in fact defined compared to the first drug experiment.
