# Supplementary material for: Epigenetic Heritability of Cell Plasticity Drives Cancer Drug Resistance through a One-to-Many Genotype-to-Phenotype Paradigm
Source: Cancer Res. 2025 Jun 11;85(15):2921–38. doi: 10.1158/0008-5472.CAN-25-0999 (PMC12314525; doi:10.1158/0008-5472.CAN-25-0999)
Supplement: Supplementary Figure 7 — Allele frequency distribution in parental vs capivasertib->trametinib treated organoids [file can-25-0999_supplementary_figure_7_suppsf7.pdf]

Supplementary Figure 7

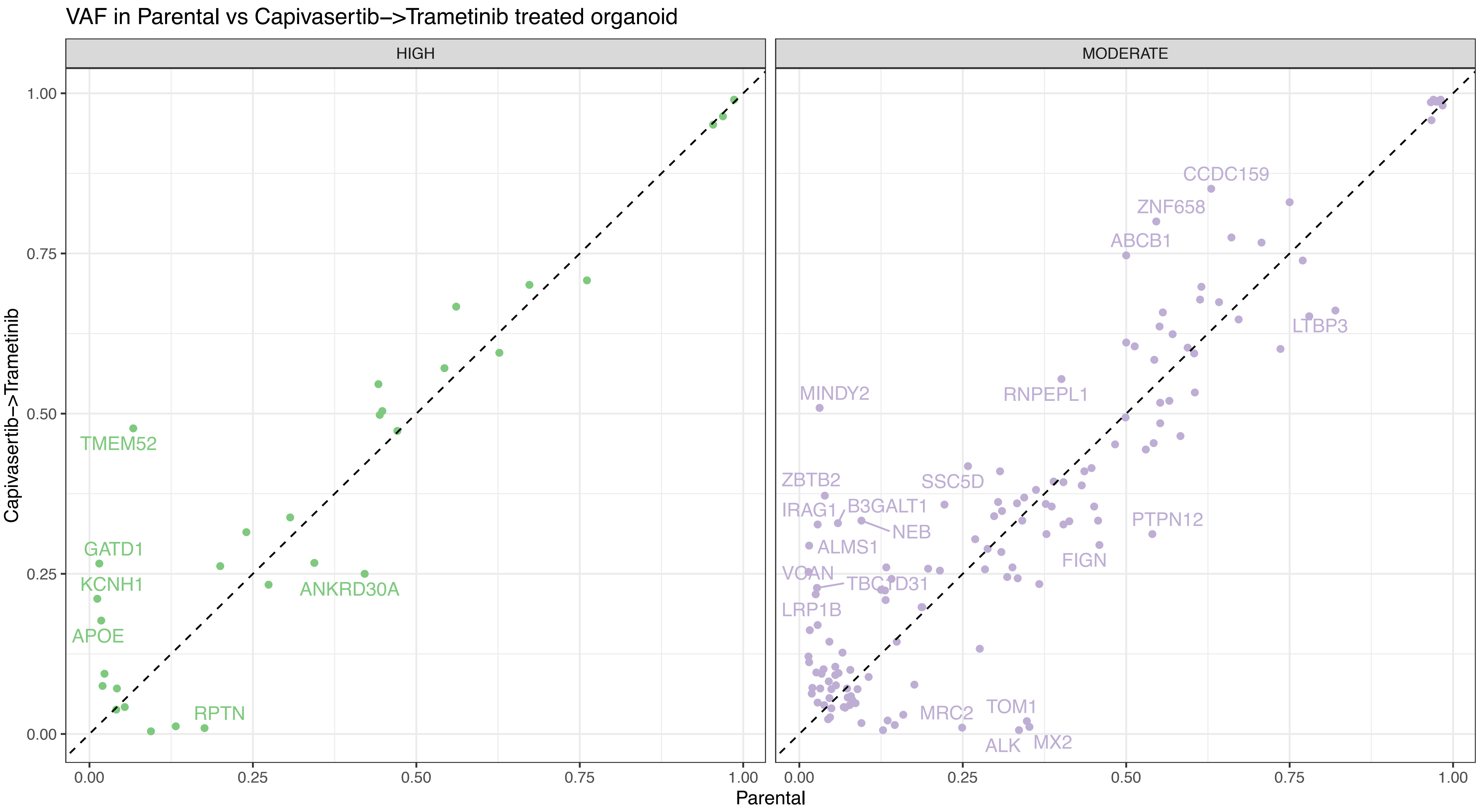


Supplementary Figure 7. Allele frequency distribution in parental vs capivasertib->trametinib treated organoids. Scatterplot of the variant allele frequency of putative functional mutations. Mutations were selected as either having VEP MODERATE impact and being either deleterious according to SIFT or damaging according to PolyPhen (left panel) or having VEP HIGH impact (right panel).
