# Supplementary material for: Epigenetic Heritability of Cell Plasticity Drives Cancer Drug Resistance through a One-to-Many Genotype-to-Phenotype Paradigm
Source: Cancer Res. 2025 Jun 11;85(15):2921–38. doi: 10.1158/0008-5472.CAN-25-0999 (PMC12314525; doi:10.1158/0008-5472.CAN-25-0999)
Supplement: Supplementary Figure 8 — Copy number profiles of treated and untreated organoids from low-pass WGS [file can-25-0999_supplementary_figure_8_suppsf8.pdf]

Supplementary Figure 8

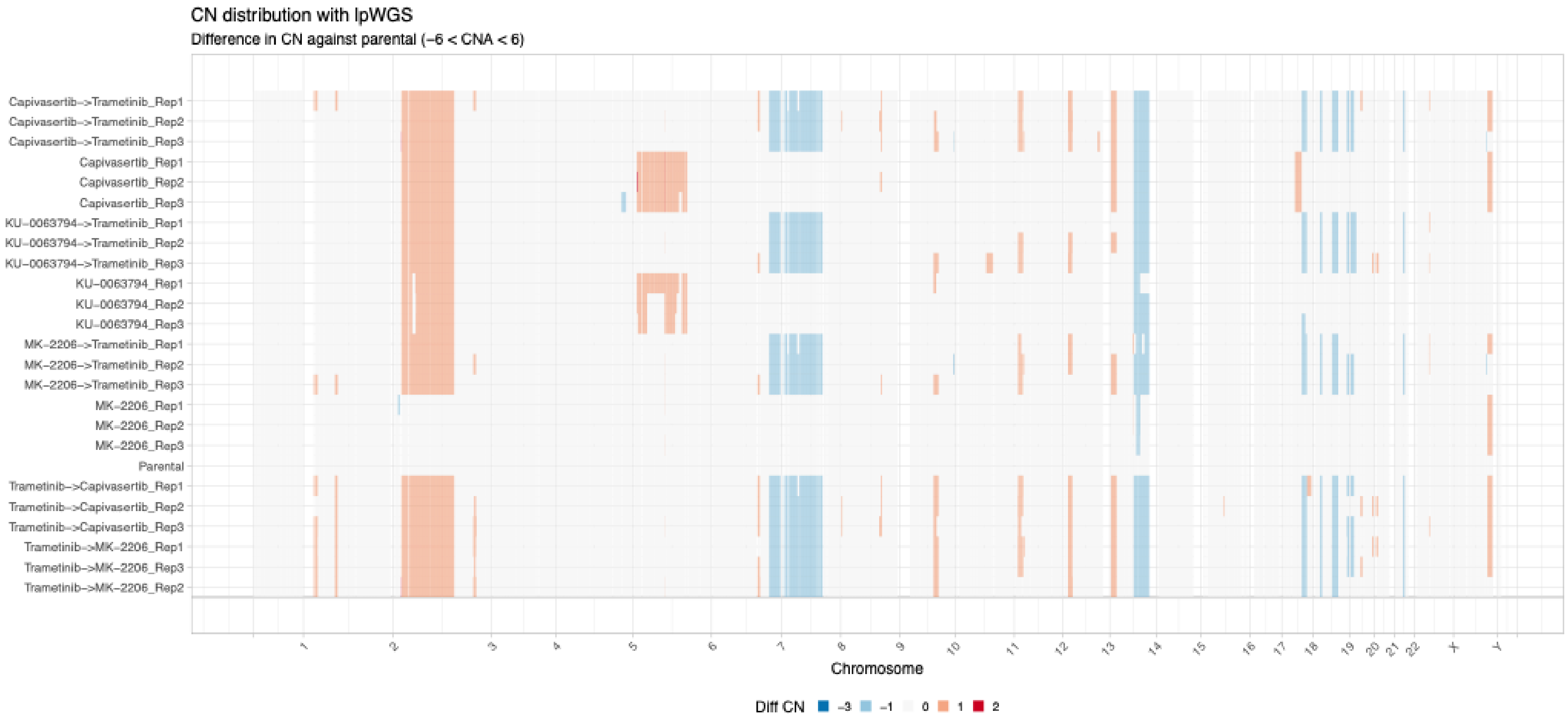

Supplementary Figure 8. ***Copy number profiles of treated and untreated organoids from low-pass WGS.*** Heatmap of relative copy number profiles for all the organoids in the experiment. Gain and losses are expressed compared to the Parental. The similarity across replicas and drug treatments is an orthogonal validation of the results obtained by lentiviral barcoding.
