# Supplementary material for: Epigenetic Heritability of Cell Plasticity Drives Cancer Drug Resistance through a One-to-Many Genotype-to-Phenotype Paradigm
Source: Cancer Res. 2025 Jun 11;85(15):2921–38. doi: 10.1158/0008-5472.CAN-25-0999 (PMC12314525; doi:10.1158/0008-5472.CAN-25-0999)
Supplement: Supplementary Figure 9 — Lentiviral barcodes distribution in 10X scRNA-seq [file can-25-0999_supplementary_figure_9_suppsf9.pdf]

Supplementary Figure 9

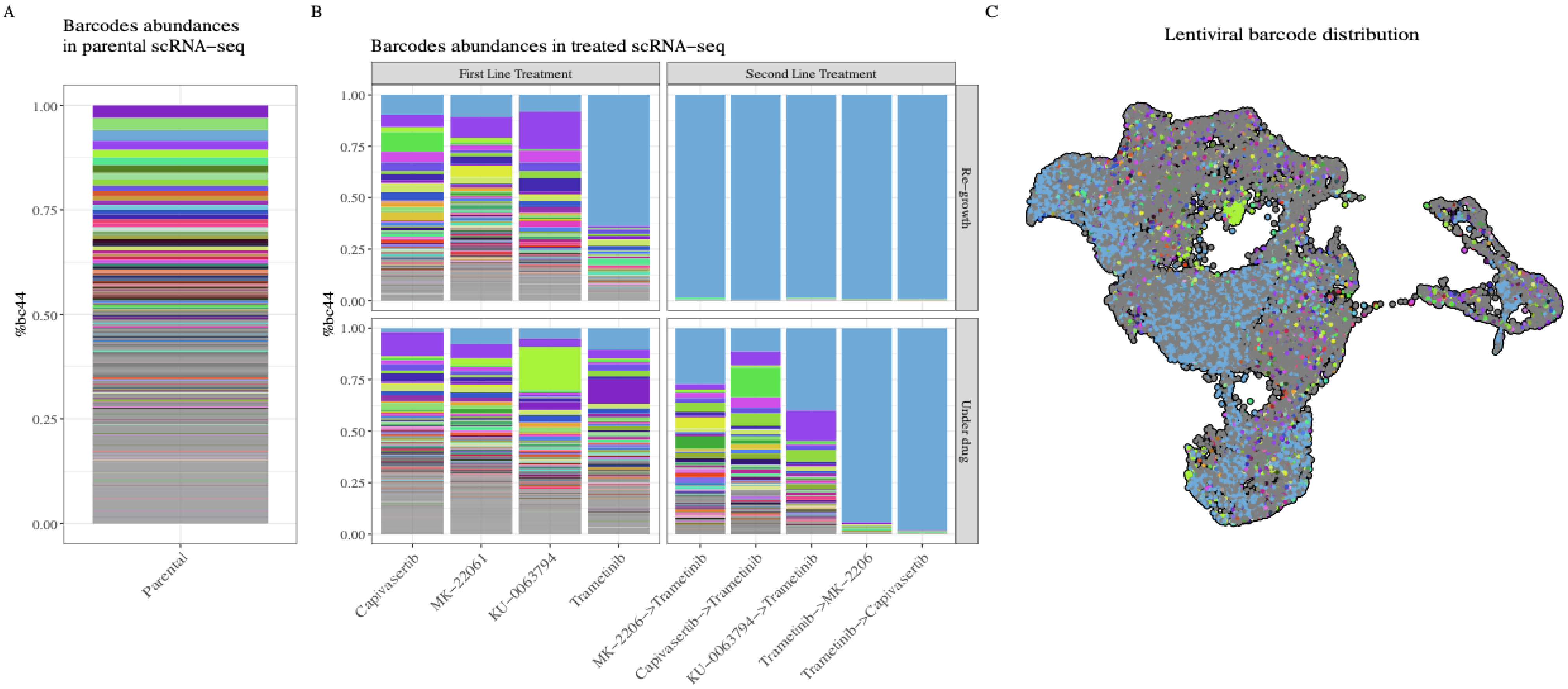

Supplementary Figure 9. ***Lentiviral barcodes distribution in 10X scRNA-seq.*** (A-B) Barcode distribution in the parental and the treated organoids respectively, the top 100 barcodes in frequency are coloured the others are shown in grey. (C) UMAP coloured by barcode as in Figure 3C. Colours are consistent with Figures 2-3 and across panel, such that each colour is always a unique barcode.
