# Supplementary material for: Epigenetic Heritability of Cell Plasticity Drives Cancer Drug Resistance through a One-to-Many Genotype-to-Phenotype Paradigm
Source: Cancer Res. 2025 Jun 11;85(15):2921–38. doi: 10.1158/0008-5472.CAN-25-0999 (PMC12314525; doi:10.1158/0008-5472.CAN-25-0999)
Supplement: Supplementary Figure 10 — Normalised expression for a set of marker genes for colon cell and UMAP feature plots [file can-25-0999_supplementary_figure_10_suppsf10.pdf]

Supplementary Figure 10

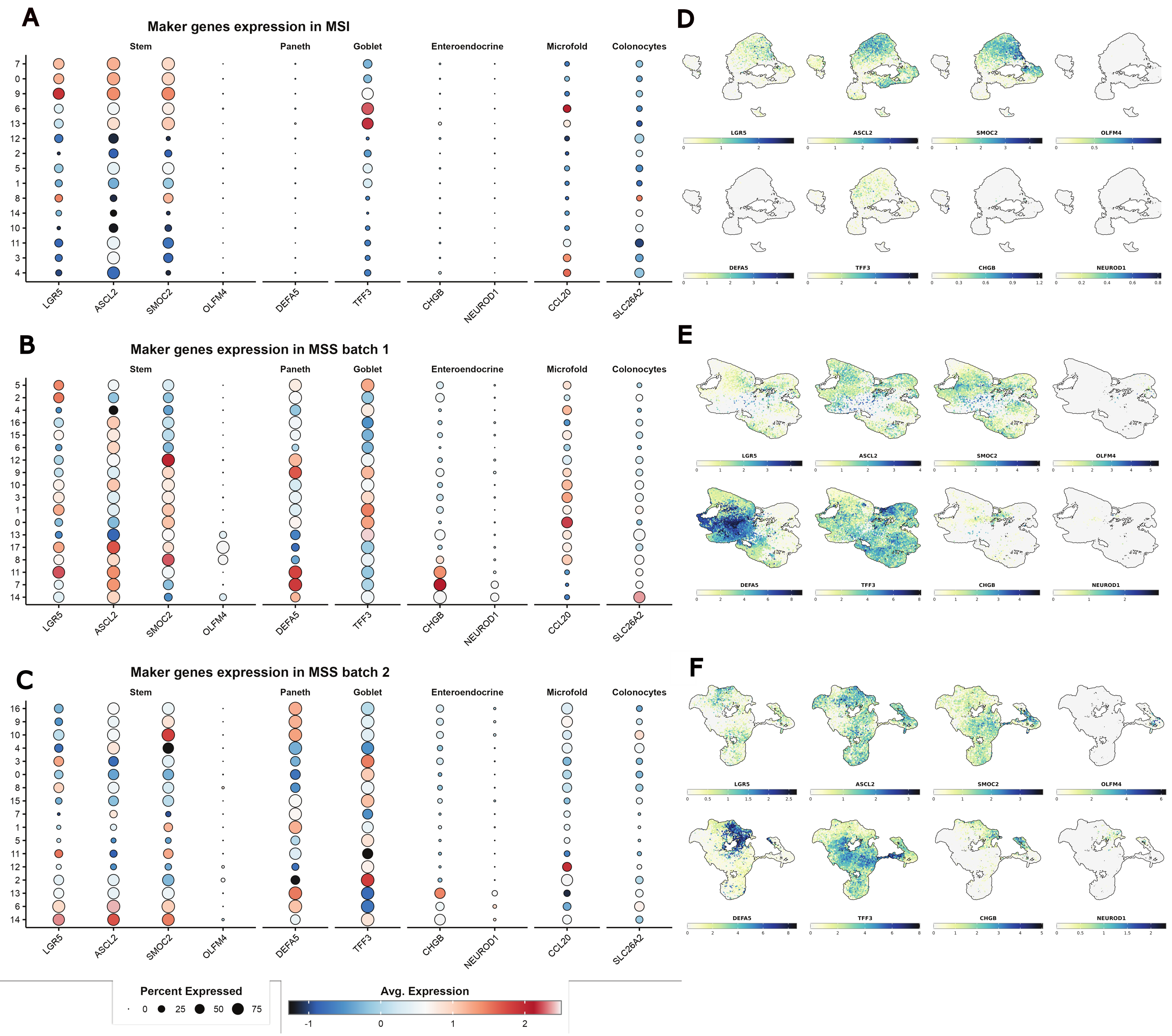

Supplementary Figure 10. (A,B,C). Normalised expression for a set of marker genes for colon cell types across Leiden clusters in MSI organoids and MSS organoid batches 1 and 2. Average expression is normalised by rows. (D,E,F) UMAP feature plots showing the spatial distribution of a subset of the markers used in panels (A,B,C) in MSI organoids and MSS organoid batches 1 and 2, highlighting cell type-specific expression patterns.
