# Supplementary material for: Epigenetic Heritability of Cell Plasticity Drives Cancer Drug Resistance through a One-to-Many Genotype-to-Phenotype Paradigm
Source: Cancer Res. 2025 Jun 11;85(15):2921–38. doi: 10.1158/0008-5472.CAN-25-0999 (PMC12314525; doi:10.1158/0008-5472.CAN-25-0999)
Supplement: Supplementary Figure 11 — Transcriptional Landscape of organoids treated with Oxaliplatin and SCH77298 [file can-25-0999_supplementary_figure_11_suppsf11.pdf]

Supplementary Figure 11

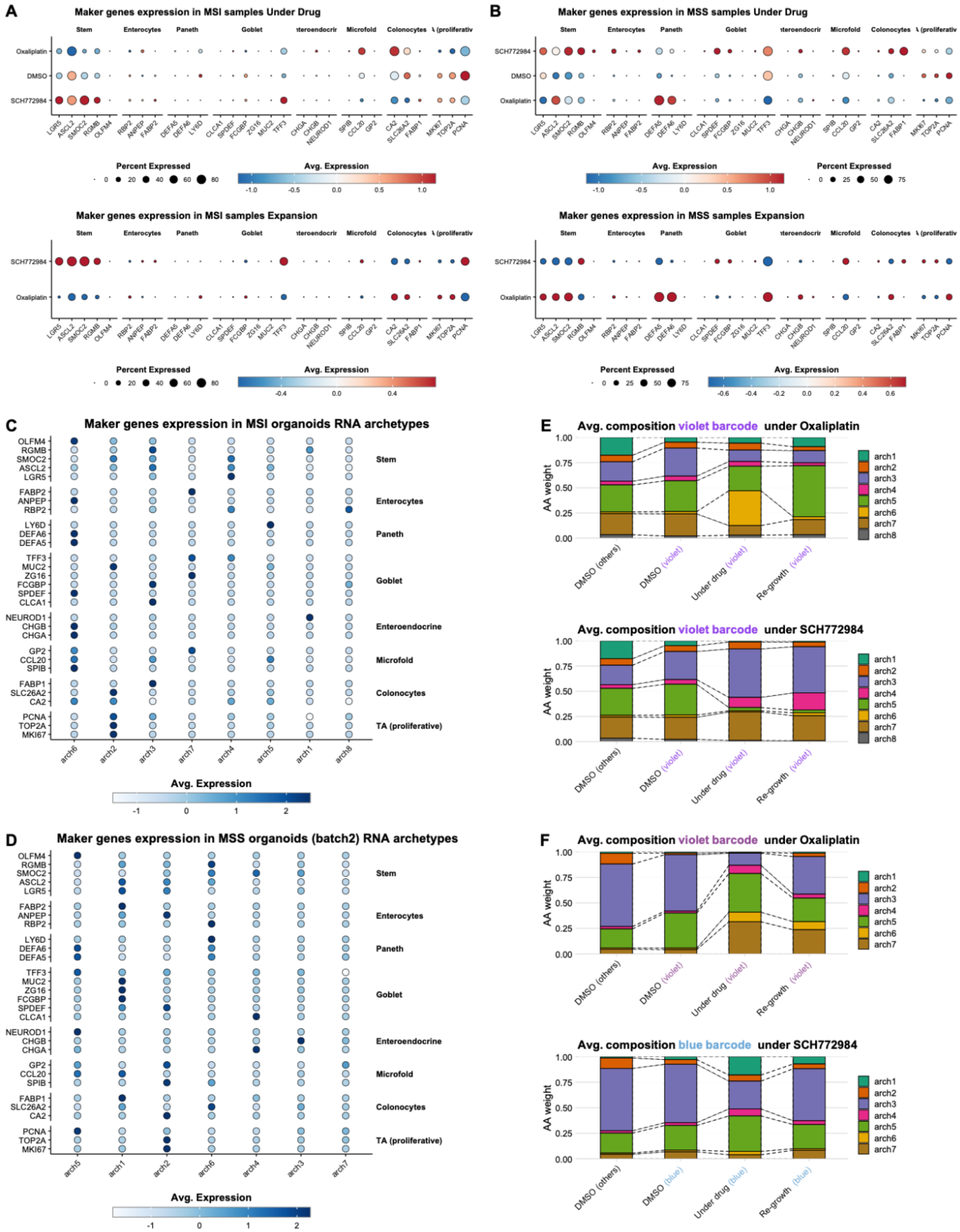

Supplementary Figure 11. Transcriptional Landscape of organoids treated with Oxaliplatin and SCH77298. (A-B) *Z-score distribution of markers in Figure 4E, we note how the MSI organoid seems to be less heterogenous.* ***(C-D)*** *Cell type specific marker expression for each archetype in the MSI and AKT (second batch) organoid, in both cases we see how archetypes capture some cell-type specific variability.* ***(E-F)*** *Archetype distribution for selected barcodes over the course of the experiment in MSI* ***(E)*** *and AKT batch 2* ***(F)*** *organoids. We find again a massive transcriptional rewiring after therapy that tends to slowly come back to an untreated like status after re-growth. It is important to note that this effect is not homogenous across drugs and organoids in this case with the AKT rapidly coming back to a DMSO like state after SCH77298, while in the MSI case it seems like the drug the is the induces this strong on-off transcriptional requiring is the Oxaliplatin.*
