# Supplementary material for: Epigenetic Heritability of Cell Plasticity Drives Cancer Drug Resistance through a One-to-Many Genotype-to-Phenotype Paradigm
Source: Cancer Res. 2025 Jun 11;85(15):2921–38. doi: 10.1158/0008-5472.CAN-25-0999 (PMC12314525; doi:10.1158/0008-5472.CAN-25-0999)
Supplement: Supplementary Figure 12 — Normalised Progeny pathway and transcriptional factor scores by archetypes [file can-25-0999_supplementary_figure_12_suppsf12.pdf]

Supplementary Figure 12

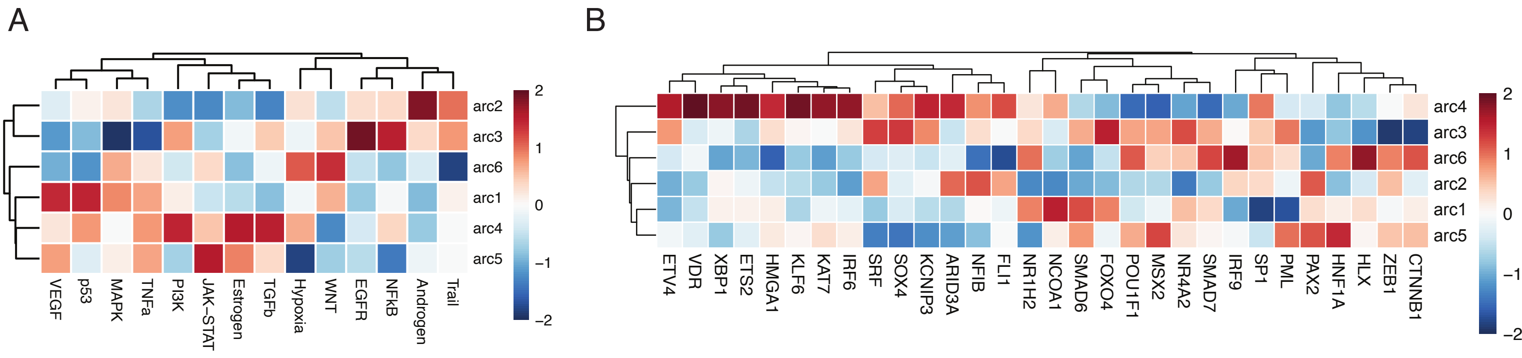

Supplementary Figure 12. A. Normalised Progeny pathway scores by archetypes. Scores are computed using a multivariate linear model on the annotated gene importance weights for each pathway B. Normalised Transcription Factors (TFs) scores by archetype for the top 30 TFs with highest variance. Scores are computed using a multivariate linear model incorporating the TF target gene expression and the direction of the correlation, i.e. inhibition or activation.
