# Supplementary material for: Epigenetic Heritability of Cell Plasticity Drives Cancer Drug Resistance through a One-to-Many Genotype-to-Phenotype Paradigm
Source: Cancer Res. 2025 Jun 11;85(15):2921–38. doi: 10.1158/0008-5472.CAN-25-0999 (PMC12314525; doi:10.1158/0008-5472.CAN-25-0999)
Supplement: Supplementary Figure 13 — Cell type and Archetype composition in TCGA Colorectal Cancer samples [file can-25-0999_supplementary_figure_13_suppsf13.pdf]

Supplementary Figure 13

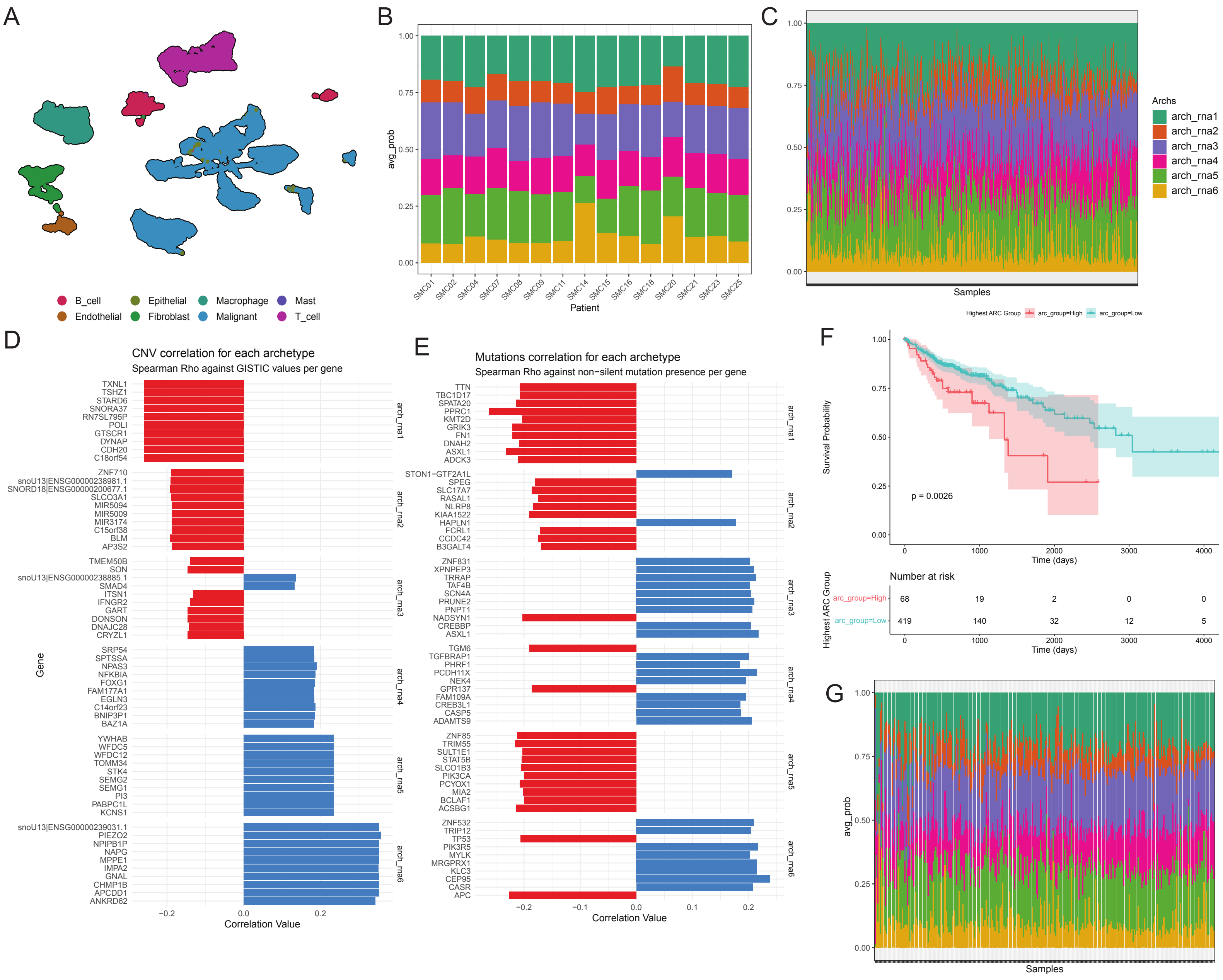

Supplementary Figure 13. (A) Cell type composition for the single-cell cancer dataset in [1]. (B) Archetype average probability by sample for the single-cell cancer dataset, after filtering for only malignant cells. (C) Archetype composition in TCGA Colorectal Cancer samples. (D) Spearman correlation between GISTIC CNV values per gene and archetype scores in TCGA. Positive correlations (in blue) i.e. the archetype score is correlated with the presence of amplification, while negative correlations (in red), the archetype is correlated with deletion. Only top 10 entries per archetype are shown. (E) Spearman correlation between mutation presence and archetype scores. Only top 10 entries per archetype are shown. (F) Kaplan-Meier survival curve for stratification by archetype 3, with the optimal cutoff determined using the R function cutpointr [4]. (G) Archetype composition in Hartwig colorectal samples.
