# Supplementary material for: Epigenetic Heritability of Cell Plasticity Drives Cancer Drug Resistance through a One-to-Many Genotype-to-Phenotype Paradigm
Source: Cancer Res. 2025 Jun 11;85(15):2921–38. doi: 10.1158/0008-5472.CAN-25-0999 (PMC12314525; doi:10.1158/0008-5472.CAN-25-0999)
Supplement: Supplementary Figure 14 — Trackplot of peaks enriched in gene promoters [file can-25-0999_supplementary_figure_14_suppsf14.pdf]

Supplementary Figure 4

Full figure at
https://figshare.com/articles/figure/Supplementary_Figure_14/29039624?file=54461525

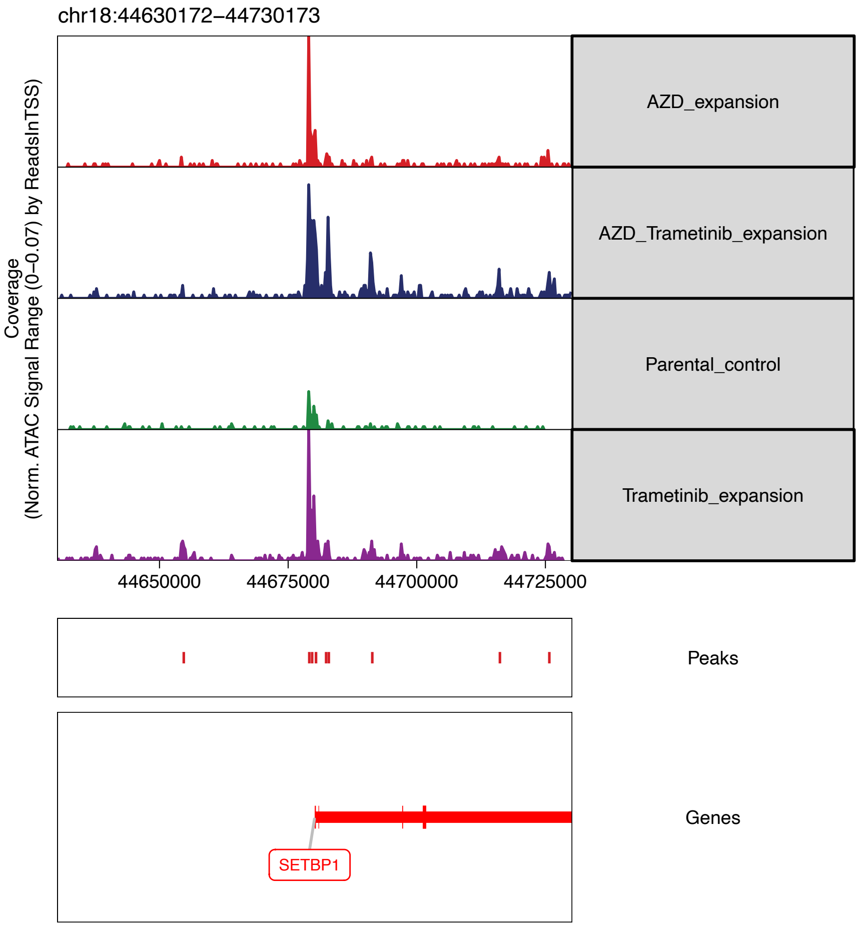

Supplementary Figure 14. Trackplot of peaks enriched in gene promoters after trametinib treatment. We first run differential expression analysis on the Multiome GEX part using a non-parametric Wilcoxon test grouping by drug. We then perform the same analysis but with peak coverage. In both cases we selected the results with FDR <= 0.01 and absolute logFC > 1. To generate the final set we intersected the significant peaks and genes, by considering only peaks in the promoter regions.
