# Supplementary material for: Epigenetic Heritability of Cell Plasticity Drives Cancer Drug Resistance through a One-to-Many Genotype-to-Phenotype Paradigm
Source: Cancer Res. 2025 Jun 11;85(15):2921–38. doi: 10.1158/0008-5472.CAN-25-0999 (PMC12314525; doi:10.1158/0008-5472.CAN-25-0999)
Supplement: Supplementary Figure 15 — Biological characterisation of trametinib resistant population [file can-25-0999_supplementary_figure_15_suppsf15.pdf]

Supplementary Figure 15

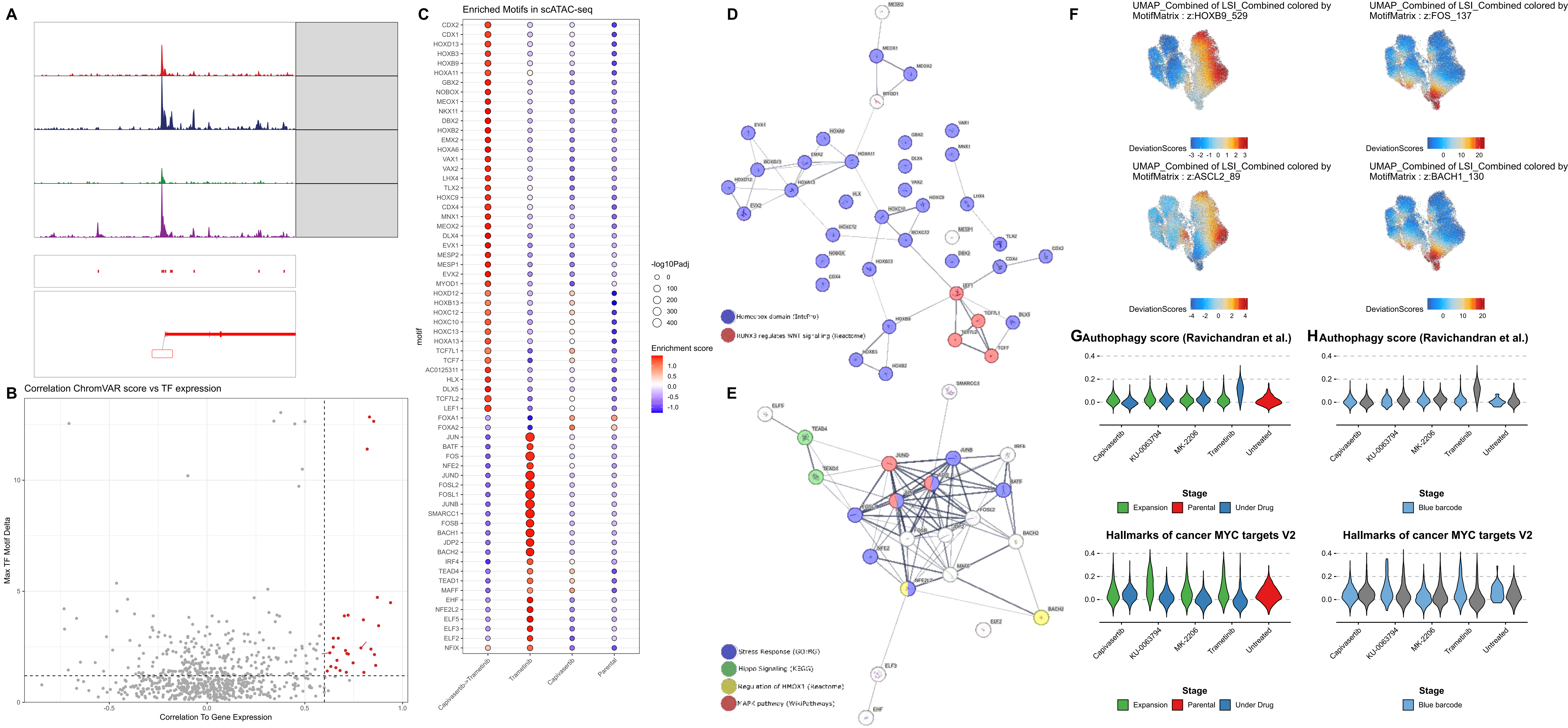

Supplementary Figure 15. Biological characterisation of trametinib resistant population. (A) SETBP1 chromatin profile in the promoter diploid region. (B) Scatterplot of Transcription Factor (TF) Motifs variance computed using ChromVAR62 from the ATAC portion of the Multiome and expression from the GEX part. Points in red are enriched TFs that also show consistent high change in expression, selected as having correlation > 0.6, adjusted p-value < 0.001 and TFs Motif variance greater than the 10% quantile. (C) Heatmap of enriched Motifs in Marker Peaks. We filtered Enriched Motifs with FDR <= 0.001 & Log2FC >= 1 (D-E) StringDB analysis of Motifs enriched respectively in capivasertib->trametinib and trametinib samples, showing involvement of TFs active in WNT and MAPK signalling pathways as well as TFs containing HomeoBox domains. Analysis was conducted using the browser version 12.0 of StringDB72 (F) UMAP plots for some of the top enriched Motifs. Sample specific localisation is evident. (G-H) Gene Module score for the autophagy signature in Ref.29 and the MSigDB Hallmark of Cancer MYC targets V260, grouped respectively by phase and barcode. The plot shows how in the under-drug phase cells under trametinib have and high level of autophagy and a correspondingly lower level of MYC activation. The blue resistant barcode however shows an opposite trend and seems to resemble the parental and re-growth behaviour even under drug. Panel (H) scores are computed for the under-drug samples.
